# Poly(lactide)-Based Materials Modified with Biomolecules: A Review

**DOI:** 10.3390/ma17215184

**Published:** 2024-10-24

**Authors:** Małgorzata Świerczyńska, Marcin H. Kudzin, Jerzy J. Chruściel

**Affiliations:** 1 Łukasiewicz Research Network—Lodz Institute of Technology (ŁIT), 19/27 Marii Skłodowskiej-Curie Str., 90-570 Łódź, Poland; malgorzata.swierczynska@lit.lukasiewicz.gov.pl (M.Ś.); marcin.kudzin@lit.lukasiewicz.gov.pl (M.H.K.); 2 Circular Economy Center (BCG), Environmental Protection Engineering Research Group, Łukasiewicz Research Network—Lodz Institute of Technology (ŁIT), Brzezińska 5/15, 92-103 Łódź, Poland; 3 Institute of Polymer and Dye Technology, Faculty of Chemistry, Lodz University of Technology, Stefanowskiego 16, 90-537 Łódź, Poland

**Keywords:** poly(lactic acid) (PLA), PLA-based materials, biocomposites, characterization, applications

## Abstract

Poly(lactic acid) (PLA) is characterized by unique features, e.g., it is environmentally friendly, biocompatible, has good thermomechanical properties, and is readily available and biodegradable. Due to the increasing pollution of the environment, PLA is a promising alternative that can potentially replace petroleum-derived polymers. Different biodegradable polymers have numerous biomedical applications and are used as packaging materials. Because the pure form of PLA is delicate, brittle, and is characterized by a slow degradation rate and a low thermal resistance and crystallization rate, these disadvantages limit the range of applications of this polymer. However, the properties of PLA can be improved by chemical or physical modification, e.g., with biomolecules. The subject of this review is the modification of PLA properties with three classes of biomolecules: polysaccharides, proteins, and nucleic acids. A quite extensive description of the most promising strategies leading to improvement of the bioactivity of PLA, through modification with these biomolecules, is presented in this review. Thus, this article deals mainly with a presentation of the major developments and research results concerning PLA-based materials modified with different biomolecules (described in the world literature during the last decades), with a focus on such methods as blending, copolymerization, or composites fabrication. The biomedical and unique biological applications of PLA-based materials, especially modified with polysaccharides and proteins, are reviewed, taking into account the growing interest and great practical potential of these new biodegradable biomaterials.

## 1. Introduction

Usually, the surface properties of materials are insufficient in terms of biocompatibility, adhesion properties, wettability, etc. Therefore, they need to be modified accordingly in order to obtain the desired surface finish and improve their functional properties [1]. A successful application in many industrial fields depends not only on mechanical properties of the materials, but also on their controlled surface properties such as hydrophilicity, the presence of reactive functional groups, roughness, etc.; especially for biomedical applications of poly(lactic acid) (PLA) when it is used in the human body, where the surface characteristics are crucial, improving hydrophilicity and introducing reactive groups are beneficial for cell affinity and cell adhesion [2,3]. Unfortunately, despite all its advantages, PLA applications are limited due to a number of disadvantages. These disadvantages include hydrophobicity and a lack of specific functional groups for cell attachment and growth, favoring cell affinity, which limits the applications in the field of tissue engineering and other biotechnological applications, requiring further appropriate surface modifications [4,5]. These reasons inspired a great number of attempts concerning the modification of chemical and physical PLA properties, as has been described in the world scientific literature. In this review, we have tried to summarize published results on this topic in recent years. It seems that modifications of PLA properties will have great potential in biomedical applications in the near future.

### Poly(lactic Acid) (PLA)

Throwing away plastic waste has caused serious environmental problems such as global warming and plastic pollution [6]. Therefore, there is an urgent need to replace petroleum-based materials with bio-based polymers [7]. Poly(lactic acid) is a polyester made up of a lactic acid building units, and, compared to other petroleum-based plastic, PLA has shown many excellent properties such as good processability and mechanical properties, making it one of the top choices among biodegradable materials [8,9]. Poly(lactic acid) is a bio-based biodegradable aliphatic polyester that can be produced from renewable resources such as sugar from beets and sugar cane, a starch from a corn and potatoes, and so forth [8,10]. Poly(lactic acid) has a wide range of uses. It is applied widely in packaging and agriculture. PLA is appropriate for biomedical industries, such as tissue scaffolds, internal sutures, and implant devices, like stents and implantable drug dispensers that are designed to biodegrade over time [11]. Because PLA has no functional groups in its structure, this limits its application in terms of affinity for cells [12].

**Figure 1:**
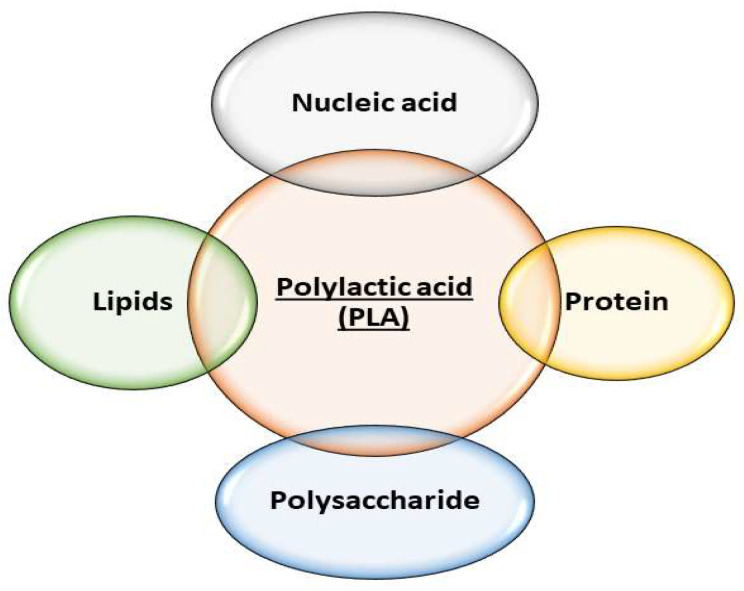
Modification of PLA with natural substrates to improve its surface properties.

Therefore, natural polymers, mainly polysaccharides and proteins, which can bind cells and provide an appropriate hydrophilic environment, thus ensuring cell attachment and proliferation, are often used for the modification of PLA properties (Figure 1) [13].

The main text of our review article is divided into three parts, which describe PLA modified with nucleic acids (in Section 2), poly(lactide) scaffolds modified with polysaccharides (in Section 3), and lipid-modified poly(lactide) scaffolds (in Section 4).

## 2. PLA Modified with Nucleic Acids

**Figure 2:**
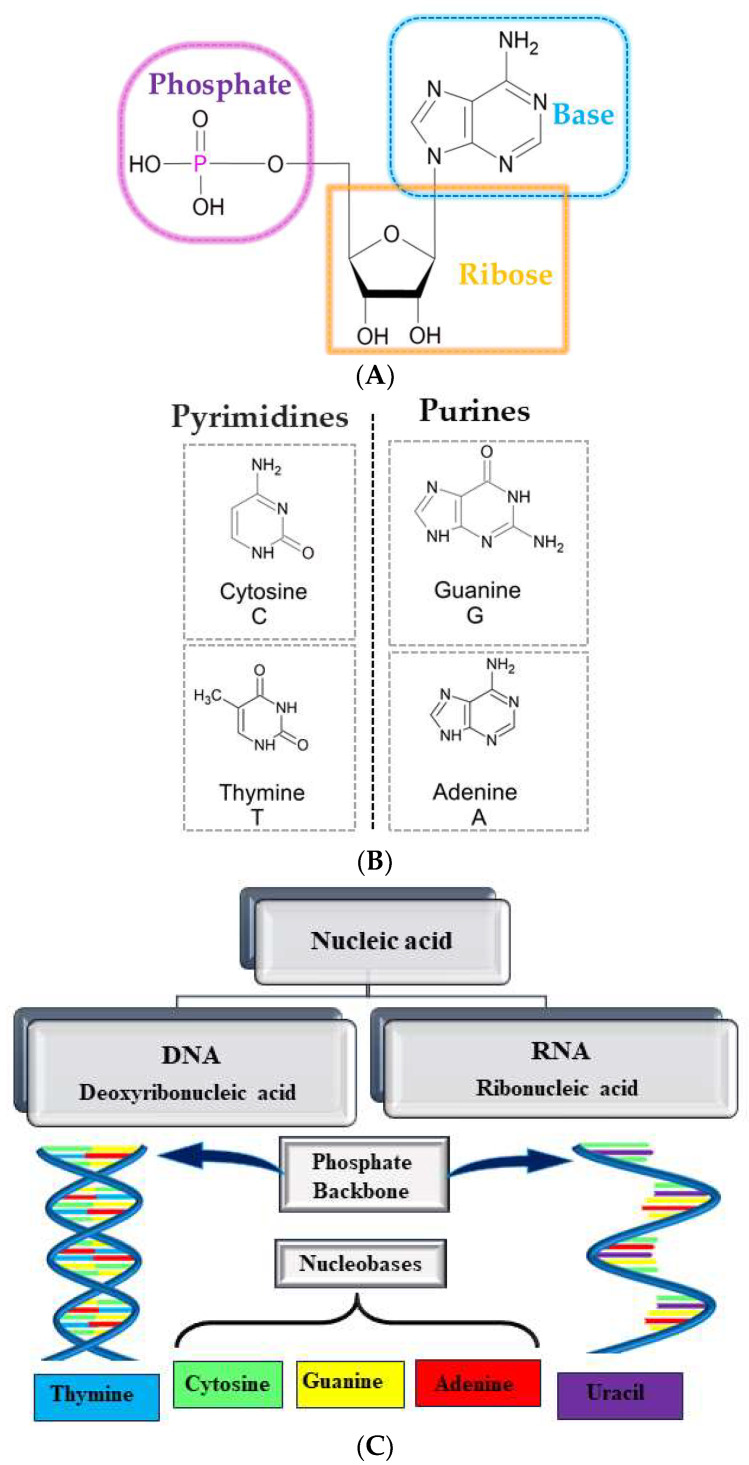
(**A**) Chemical structure of a DNA nucleotide, composed of a sugar, a phosphate group, and a base. (**B**) The chemical structures of pyrimidines (cytosine and thymine) and purines (guanine and adenine). (**C**) Nucleic acids (RNA and DNA), which are present in all living organisms.

There has been an increase of interest in nanomedicine as it has unprecedented potential in cancer diagnosis and therapy. The nucleic acids [e.g., deoxyribonucleic acids (DNA) and ribonucleic acids (RNA)] (Figure 2C) added to nanomedicines are particularly interesting from a medical point of view. These organic chemical compounds are made of monomeric units called nucleotides, which consist of a nucleoside (a pentose sugar linked to a nitrogenous base) and a phosphate moiety. A double helix is formed from two complementary strands of nucleotides held together by hydrogen bonds between two base pairs: pyrimidines [guanine (G) and cytosine (C)] and purines [adenine (A) and thymine (T)]. The chemical structure of DNA is presented in Figure 2A, and the chemical structures of the nitrogenous bases in Figure 2B. DNA mainly serves as a carrier of genetic information, while RNA can play various roles in the functioning of the cell, modulating the expression of target genes or proteins.

PLA is one of the most widely used polymers for drug delivery applications due to its attractive mechanical and processing properties [14,15]. PLA-based systems have been developed to deliver a variety of payloads, from small drug molecules to nucleic acids and large proteins, acting in a sustained-release manner [16]. As a biocompatible polymer, PLA produces safe and non-toxic degradation products, making it a good candidate for many pharmaceutical and medical applications [17]. Biodegradation involves a hydrolytic degradation of PLA to lactic acid, which is eliminated from the human body as CO_2_ and H_2_O [18]. The carrier delivery system using biodegradable PLA is very important for controlled release. Drug release kinetics is a result of polymer degradation as well as drug diffusion through the polymer matrix during its degradation and erosion [19].

PLA materials modified with nucleic acids can deliver drugs, genes, and proteins in a controlled and stable manner and increase their therapeutic effectiveness. This method of modifying PLA may lead to the discovery of new materials and open up new research opportunities that may contribute to the development of this field of biomedicine. The uniqueness of PLA as a material lies in its ability to adjust its chemical, physical, and mechanical properties, controlling its degradation rate [14,15,20,21]. Through modification with nucleic acids, PLA can perform various functions and have different mechanisms of action. PLA modified with nucleic acids shows very interesting properties. Because PLA is a biodegradable and biocompatible polymer, it can be used in the biomedical field, among others, in the delivery of drugs or genes and can be used in tissue engineering [22]. Due to the low cell adhesion of PLA, it is biologically inert, and is characterized by a slow degradation rate, and these features limit its applications [23]. Therefore, one of the methods of modifying PLA is the use of nucleic acids, which can act as ligands for specific receptors on the surface of or inside the cell.

Microparticles or nanoparticles (NPs) as biological carriers or therapeutic agents can be encapsulated in polymer particles for localized and systemic delivery [24]. A drug delivery system (DDS) based on PLA has many advantages, including targeted delivery and sustained drug release, and it also increases the stability of encapsulated biopharmaceuticals for enzymatic degradation [25,26]. In recent years, researchers have particularly focused on the delivery of anticancer drugs [27,28] and those targeted at a specific organ, mainly the brain [29,30,31]. Gene therapy is of great interest to researchers because it has the potential to combat genetic diseases [32], becoming a promising therapy, especially in replacing standard methods of treating complex diseases, including cancer, hereditary diseases, or chronic infections. Nucleic acids can be applied that can introduce genes that are functional and lead to effects following the disease, and can also cause toxic effects by contributing to blocking the translation of mRNA. The use of nucleic acids involves many obstacles, like the occurrence of nucleases, biological barriers, endocytosis, as well as the instability of the nucleic acid molecules. To achieve a solution and overcome the above obstacles, nanotechnology is used, which incorporates the nucleic acids into various nano- and microparticles [33,34].

Being safe for humans and having versatile biomedical applications, PLA has also been used in gene delivery applications [35]. In gene therapy, the therapeutic deoxyribonucleic acid and the ribonucleic acid gene molecules used were capable of changing the defective genes, modifying the missing genes, and silencing the mutated genes [36]. With the growing understanding of disease-causing genes, gene therapy has become a new hope in the treatment of a number of currently incurable diseases [37,38,39].

Jain et al. created new composite nanoparticles ensuring high transfection efficiency of cationic peptide-DNA NPS combined with the biocompatible poly(lactic acid) and poly(ethylene glycol) (PLA-PEG). The cationic peptide was enclosed in the PLA-PEG copolymer matrix and was used to condense DNA into NPs. The obtained material was characterized by excellent physicochemical properties and a high encapsulation efficiency. A differentiation of the copolymers affected the DNA release rate. In this way, a group of researchers demonstrated the production of composite NPs for controlled DNA delivery [40].

The application of small interfering RNA (siRNA) is a potential therapeutic strategy for many diseases, including those of the respiratory system and the nervous system, autoimmune diseases, and cancers, due to highly specific and effective gene silencing [41,42]. Unfortunately, the biomedical applications of siRNA have encountered a number of obstacles, including the rapid degradation of nucleases in serum, lack of a targeting ability or a poor cellular uptake, etc. [43,44,45]. The therapeutic applications of siRNAs are limited in clinical practice due to the lack of safe and effective in vivo carriers. The group of Zhao et al. synthesized amphiphilic biodegradable triblock copolymer mPEG_2000_-PLA_3000_-*b*-R_15_ by coupling cationic polyarginine salt (R_15_) with a mPEG_2000_-PLA_3000_ copolymer. They confirmed that self-assembled nano-micelles were an effective and efficient nanocarrier for the delivery of the therapeutic siRNA in vivo. Moreover, this material is very safe because it has no toxicity or innate immune reaction. mPEG_2000_-PLA_3000_-*b*-R_15_-based polymeric nano-micelles are a promising material for therapeutic applications due to their ability to deliver hydrophobic anticancer drugs via siRNA and functionally modify micelles on the cell surface [46].

The development of microRNA-based drugs and their action strategy remains a challenge. Significant difficulties associated with their therapeutic use are related to the shortcomings of the siRNA methodology. The use of miRNA is a better single-shot multi-target gene therapy tool than that of siRNA currently used in the clinic. Combined chemotherapy and miRNA treatment may be a real strategy to increase chemotherapy sensitivity [47,48,49]. Unfortunately, the lack of effective co-delivery vehicles and development-limiting genes remains a challenge in the market. Qian et al. obtained amphiphilic star copolymers containing PLA and poly(dimethylaminoethyl methacrylate) (PDMAEMA) having AB_3_, (AB_3_)_2_, and (AB_3_)_3_ branched structures. The ability to condense miRNA and the physicochemical properties of star copolymers were characterized. Studies confirmed that the amphiphilic star-branched copolymers could have great applications in combinatorial gene delivery and hydrophobic therapeutics [50].

Nanomedicines have great potential in the effective treatment of cancer, especially when nucleic acids are used as nanomedicines in cancer therapy, because, considering their advantages such as structural programmability and versatile therapeutic functions (e.g., short hairpin RNA (shRNA), antisense DNA, immunomodulatory DNA/RNA, and DNA binding chemotherapeutics), they are excellent platforms for the co-delivery of nucleic acid drugs and chemotherapeutics in cancer therapy, which provides a new opportunity in cancer research [51,52,53,54]. Nanomedicines based on nucleic acids may constitute an excellent platform for co-delivery with other synergistic drugs used in combined anticancer therapy [55,56].

Ni et al. developed a new nanoplatform (PLA)@poly-shRNA/doxorubicin (Dox), which used poly(lactide) to form DNA-PLA micelles. PLA cores were simultaneously loaded with hydrophobic Dox for co-delivery with shRNA. They developed a hybrid nucleic acid-polymer formulation that co-delivered nucleic acid (shRNA) drugs with chemotherapeutics (Dox). The new nanomaterial contributed to a robust combined therapeutic efficacy in cancer therapy [57].

## 3. Poly(lactide) Scaffolds Modified with Polysaccharides

The use of materials of both synthetic and natural origin as components of biomaterials is constantly developing. The synthesis of unique chemical structures can provide specific functions for desired applications, improve the range of possible biomaterials, and also increase their biocompatibility [58,59]. Biomolecules such as natural polysaccharides are also used as biomaterials in various applications, including as drug carriers and scaffolds in tissue engineering [60,61]. Biocompatible polymers are extremely useful because they do not interact specifically with biological systems, making it difficult if interactions are desired to manipulate biological responses, such as growth factor binding or enzymatic degradation [62,63]. Natural polymers or biopolymers are created by long chains of monomers of the same type or combinations of different ones. Polysaccharide biopolymers are characterized by complex secondary structures that play several roles in plants, animals, and microorganisms [64]. Their versatility and biodegradability make some of them widely used in various industries as sustainable and renewable materials, including pharmaceutical, biomedical, food, and packaging uses [65]. Biomolecules such as polysaccharides, proteins, and nucleotides are essential components and are responsible for many processes in biological systems, including cellular communication, adhesion, and molecular recognition in the immune system [66,67]. Polysaccharides are one of the main classes of biopolymers (carbohydrates) that perform various biological functions [68].

**Figure 3:**
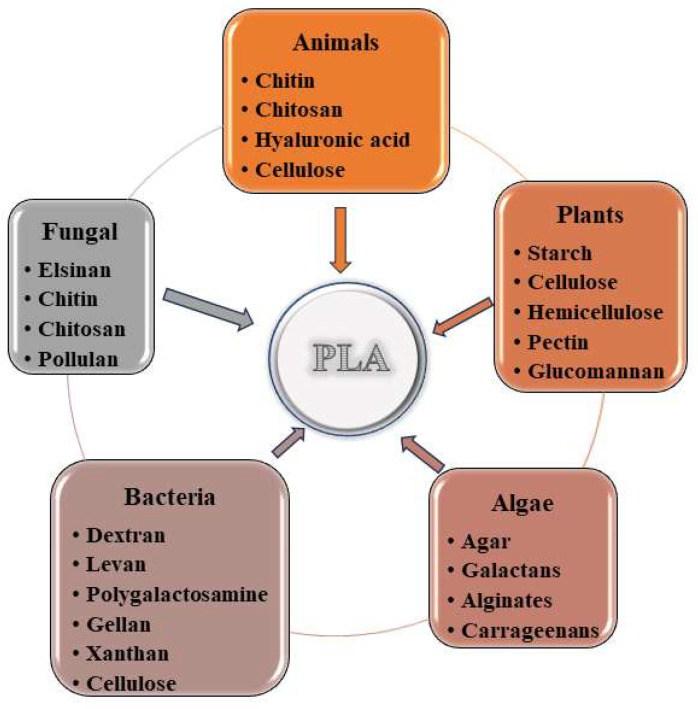
A list of natural polysaccharides used for the modification of PLA.

Due to their characteristic properties, polysaccharides can be divided into: neutral polysaccharides (dextran, cellulose, starch, pullulan, β-D-glucans, etc.), acidic (including hyaluronic acid, alginic acid, etc.), basic (chitin, chitosan, etc.), and sulfated polysaccharides (dermatan sulfate, heparin, chondroitin sulfate, fucoidan, etc.) (Figure 3) [69]. Various sources of polysaccharides make them biomolecules that can be chemically modified and can be used in various medical and non-medical fields [70,71].

Blends of PLA with non-acetylated soda lignin (SL) and acetylated soda lignin (ASL) were extruded giving antioxidant PLA)/lignin composites. The PLA/ASL composites displayed higher mechanical properties than PLA/SL composites. After lignin acetylation, good compatibility was observed between PLA and lignin. The antioxidant properties, cytocompatibility, and hemocompatibility of lignin/PLA composites might be useful for their potential biomedical applications [72].

### 3.1. Chitosan

The technique of combining polymers is increasingly used by scientists to develop new materials that exhibit properties that cannot be achieved using a single polymer [73]. In recent years, many efforts have been made to develop new polymeric materials containing PLA and chitosan as the bioactive material.

**Figure 4:**
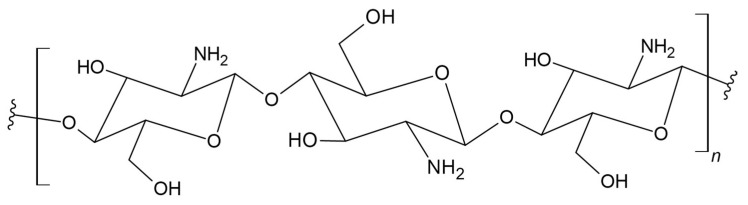
The chemical structure of chitosan.

Chitosan is obtained in the alkaline deacetylation reaction of chitin and is a copolymer of N-acetylglucosamine and D-glucosamine (Figure 4). It is characterized by solubility, unlike chitin, and it is considered as an antimicrobial polymer. Because chitosan-based materials are susceptible to moisture [74,75,76], it is useful to combine this polysaccharide with a more moisture-resistant polymer, while maintaining the overall biodegradability of the product.

Suyatma et al. described the grafting copolymerization of poly(lactic acid) on chitosan chains using two different methods. The first method involved the direct grafting of lactic acid onto chitosan using *para*-toluenesulfonic acid as a catalyst. The second method was based on the ring-opening polymerization (ROP) of lactide using triethylamine as the catalyst. The obtained materials have great potential for use as packaging material or as a compatible agent in chitosan/PLA blends [77]. Recently, Bonilla et al. prepared films based on poly(lactic acid) (PLA) and various amounts of chitosan powder by extrusion. It was found that the addition of chitosan had no effect on the thermal properties of PLA. However, the obtained material was characterized by higher water vapor permeability than the PLA material itself. Additionally, the prepared composite material showed significant antimicrobial activity [78].

Hui et al. obtained a biocomposite material, based on poly(lactic acid) (PLA) and chitosan biopolymers, which was processed by 3D printing and was useful for bone repair. They proved that biocomposite filaments consisting of PLA and 10 wt.% of chitosan can be processed by 3D printing. This new material exhibited antibacterial properties, mechanical strength, and biodegradability of the PLA/chitosan biocomposite for 3D printing, which can be useful for bone repair [79].

Abifarin et al. investigated the mechanical properties of a 3D-printed PLA/chitosan composite. They underlined that important parameters in the production of the mechanically improved material were: high filling density, a small amount of chitosan, and a lower processing temperature, in order to obtain improved, mechanically printed chitosan/PLA scaffolds [80].

Liu et al. prepared fibrous scaffolds with various proportions of PLA and chitosan, using conventional electrospinning. After cross-linking with glutaraldehyde vapor, they examined the structure, mechanical properties, hydrophilicity, and chemical interactions of the obtained scaffolds in the fibers. The PLA/chitosan fibers showed great potential for cardiac tissue engineering and accelerating myocardial regeneration [81].

### 3.2. Pullulan

The versatility of PLA is an undeniable feature of this polymer. Its attractiveness is related to its bioabsorbability, renewability, and easy repeatability [82]. In recent years, research has been focused on overcoming the limitations related to the lack of functional groups and thus increasing the applications of PLA [83,84].

**Figure 5:**
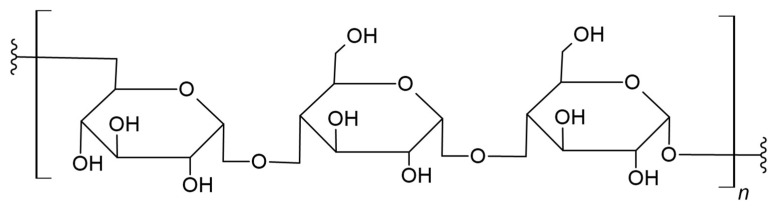
The chemical structure of pullulan.

Pullulan is a linear polysaccharide that consists of glucose units and is often described as *alpha*-1,6-conjugated maltotriose (Figure 5) [85]. Moreover, it is hemocompatible, non-immunogenic, non-carcinogenic, non-toxic, non-irritating, and biodegradable [86,87]. Due to the configuration of the monomer and the bonds present in its structure, it has many beneficial and unique properties, including biocompatibility and a hydrophilic character, which make it useful in many applications [88]. Moreover, it has good adhesive properties and is capable of forming fibers [89]. PLA has the potential for further modifications due to its molecular structure, which provides reactive sites such as hydroxyl groups, thanks to which new bonds can be formed with other compounds, which may result in new functions of the material [90].

Tang et al. developed an innovative method for the synthesis of a poly(*D*,*L*-lactide)-*graft*-pullulan (PL) copolymer using a microwave field. Microwave-assisted synthesis showed higher conversion and polylactide yield [91]. Based on this work, the research group of Xu et al. synthesized poly(lactic-*co*-glycolic acid)-*graft*-pullulan (PPLGA), thereby shortening the copolymerization time. The obtained material was characterized by self-assembly, creating thermoresponsive nanoparticles. As a result, researchers obtained a material with the thermoresponsive properties of PPLGA, which can release a thermo-sensitive drug, demonstrating itself as a material suitable for clinical applications in cancer treatments such as thermochemotherapy [92].

### 3.3. Xanthan

The coatings market includes polymers derived from petrochemical products with hazardous volatile organic compounds, which raises concerns about their safety. Therefore, instead of petrochemical polymers materials of biological origin are used for this purpose. The advantages of PLA are easy availability, low cost of acquisition, and lack of toxicity, which makes it a promising biopolymer with growing interest among scientists in replacing non-biodegradable polymers, which is a very interesting prospect because the disposal process could be carried out using various environmentally friendly methods [93].

**Figure 6:**
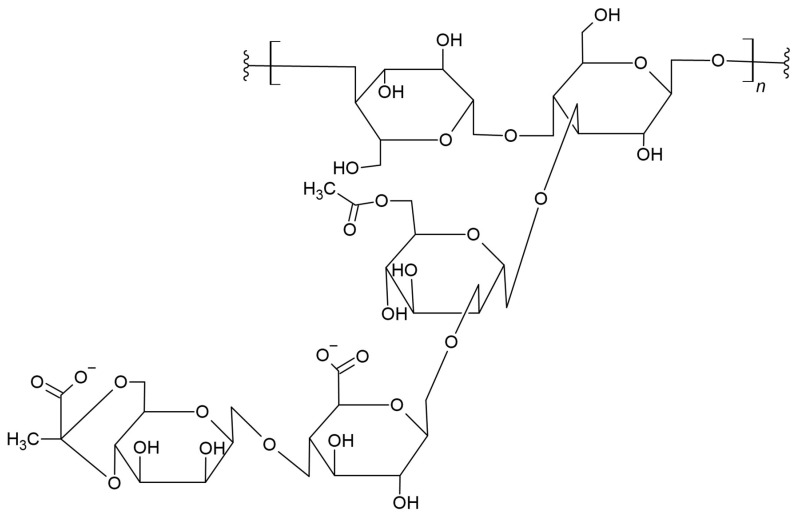
The chemical structure of xanthan.

Xanthan gum (XG) is the biodegradable branched polysaccharide consisting of a β-(1,4)-D-glucopyranose glucan backbone with side chains with -(3, 1)-α-linked D-mannopyranose-(2, 1 acid)-β-D-glucurone-(4, 1)-β-D-mannopyranose on alternating residues (Figure 6) [94,95]. It has wide biomedical and industrial applications, i.e., packaging, cosmetic, and engineering applications [96]. Xanthan gum is a non-toxic biopolymer that creates hydrocolloid solutions with high stability [97]. Due to the processing and mechanical performance of xanthan gum, the obtained biomaterials based on it still require further modifications to adapt the material to its uses [98].

The aim of the work by Abdenour et al. was to develop new, stable aqueous PLA emulsions containing xanthan gum as a thickening agent for a coating paper. The final product of biological origin was the oil-in-water (O/W) emulsion. The thickened PLA emulsion with various amounts of xanthene gum was applied as a coating to the paper, which improved the barrier properties of the base paper. The results of air and water vapor permeability tests showed that the developed PLA coating can achieve excellent overall barrier properties in combination with smooth surfaces, which is very important in the production of coated paper products in the paper industry [99].

Buoso et al. examined aqueous dispersions of poly(lactic acid) intended for use in coatings with xanthan gum (XG) serving as a thickener modulating the viscosity of the preparations. The results of the rheological analysis indicate that the viscosity increased with the increase in the concentration of the xanthan gum in the prepared dispersion. The analysis of the rheological properties of PLA/XG preparations was a very important turning point for estimating the potential of using these colloidal dispersions as new coatings in various application areas, as well as the potential use of XG in new application areas [100].

### 3.4. Gellan

Gellan is an exopolysaccharide of microbial origin produced by *Sphingomonas elodea* and *Shingomonas paucimobilis*. Due to its versatile properties, this polymer has become one of the most famous and used materials used on an industrial scale with high repeatable quality [101,102]. Due to its biocompatibility and biodegradability, interest in this polysaccharide has increased significantly and it has the potential to play several key roles in many fields, including in the biomedical, pharmaceutical, and tissue engineering fields, among others. In recent times, remarkable progress has been made in the development of materials to ensure the desired use for various purposes of application, which includes gellan [103].

**Figure 7:**
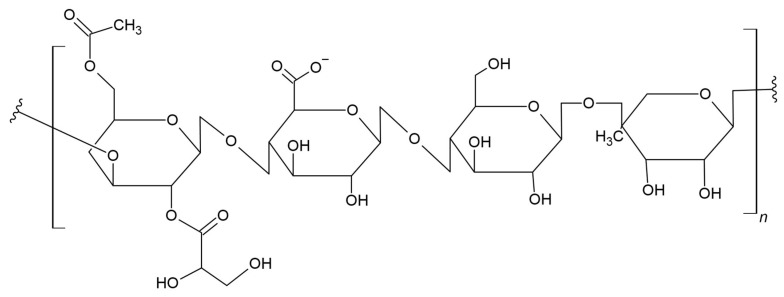
The chemical structure of gellan.

Gellan is an anionic polysaccharide with a linear structure that includes a tetrasaccharide unit of two glucoses (β-D-glucose), glucuronic acid (β-D), and a repeating rhamnose unit (l-rhamnose-α) (Figure 7) [103,104]. Some researchers use numerous hydroxyl groups and free carboxyl to improve the physico-chemical-biological properties of gellan, resulting in the continuous development of materials based on it.

Hu et al. combined the excellent mechanical properties of PLA with the biocompatibility and bioprintability of double-network gellan gum-poly(ethylene glycol) hydrogel (GG-PEGDA). The structure of the obtained PLA/GG-PEGDA scaffold was loaded with cells for intervertebral disc regeneration, using 3D bioprinting technology. The mechanical strength and degradation rate of the obtained hybrid scaffold can be adjusted according to the application requirements targeted at specific organs/tissues. The materials had great potential due to their positive biocompatibility, showing excellent ability to spread and proliferate cells. In addition, appropriate mechanical properties make it a potential platform for regulating cell functions and treating diseased tissues [105].

Hernández-García et al. prepared a two-layer foil made of cassava starch with a gellan gum and PLA-poly(3-hydroxybutyrate-*co*-3-hydroxyvalerate), PHBV copolymer. The gellan gum improved the mechanical properties of the foil and also reduced water vapor permeability. Moreover, taking into account the functional properties and adhesion of layers, the obtained material turned out to be a very good option in food packaging applications [106].

### 3.5. Carrageenans

PLA is a linear thermoplastic aliphatic polymer, characterized by biocompatibility and biodegradability, which makes it completely safe in various applications. Unfortunately, its disadvantages include brittleness and stiffness which limit its use. Therefore, efforts are still being made to improve its properties by mixing it with other additives to create a new material with unique properties [107,108,109]. Commercially produced biopolymer films have certain limitations resulting from their mechanical properties, as well as due to the high sensitivity of a humid environment [108,110]. Due to the above limitations, mixtures of materials are being developed to improve or add new properties depending on the applications.

**Figure 8:**
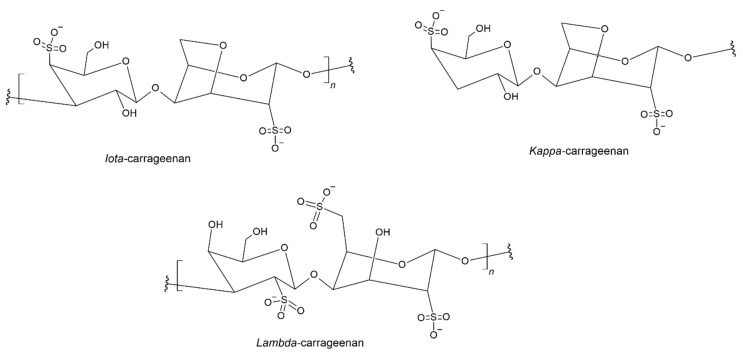
The chemical structures of carrageenans.

Carrageenans are linear typical polysaccharides that are extracted from various types of red algae from the class *Florideophyceae* [111]. This natural polymer dissolves well in water and has gelling properties. It is widely used in the food and cosmetics industries. The biological activity of carrageenan is closely related to its structure, i.e., the number and arrangement of sulfate groups [112]. The carrageenan molecule consists of 1,3-linked β-D-galactose and 1,4-linked α-D-galactose units. Carrageenan derivatives and structures present in various natural products are shown in Figure 8 [113].

Rhim developed a nanocomposite film by composing a polymer layer consisting of agar and κ-carrageenan with a layered silicate nanoclay [114]. Due to the still poor waterproof properties of the material, they were mixed with water-resistant PLA, which has good mechanical properties while maintaining the biodegradability of the material. Rahim et. all constructed multilayer nanocomposite films by combining the properties of polymers to create one structural layer. Multilayer materials consisting of PLA and agar/*κ*-carrageenan/clay nanocomposites were characterized by a higher tensile strength and thermal stability. Moreover, the tests confirmed that the water vapor permeability, water absorption coefficient, and water solubility of the obtained nanocomposite films are higher than those of films made of PLA alone [115].

### 3.6. Levan

To improve the properties of a material composed of only PLA, copolymerization, mixing, and plasticization methods were used [116]. Levan is a widely used additive with emulsifying and stabilizing properties. It can be used as a biomaterial with compatibility in drug delivery [117]. Thanks to its properties, it is used in the food industry [118]. Levan serves, among others, as a low-calorie sweetener and can replace fat [119]. Due to its similarities to a hyaluronic acid, levan has also been used in skin care products [120].

**Figure 9:**
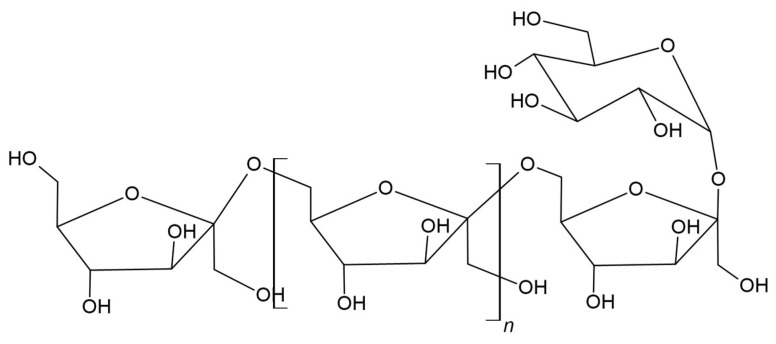
The chemical structure of levan.

Levan is an extracellular polysaccharide composed mainly of D-fructose units connected by β (2→6) glycosidic bonds, and may also contain branched β (2→1) and terminal glucose residues (Figure 9) [121]. In nature, it is synthesized from sucrose by microorganisms and several species of plants. The levan produced by bacteria has a high molecular weight of over 500,000 Da [122]. Its macromolecules are distinguished by high thermal resistance and a lack of toxic effects, which makes them suitable for use in various fields such as food, medicine, and nanotechnology [121,123,124,125].

The melting point of PLA in levan/PLA films decreased slightly with the increase in levan content [126].

### 3.7. Cellulose

**Figure 10:**
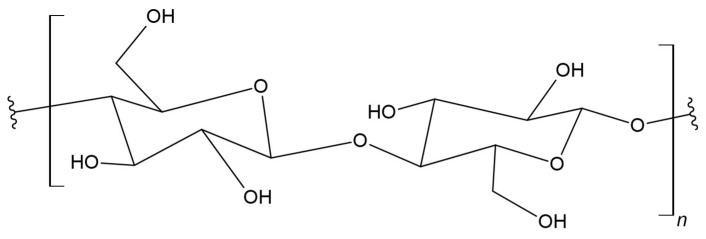
The chemical structure of cellulose.

Cellulose is an unbranched polysaccharide macromolecule consisting of β-D-glucopyranose (C_6_H_12_O_6_) units connected by β-1,4-glycosidic bonds (Figure 10). The length of β-(1,4) glucan chains varies and depends on a cellulose source [127]. The structural unit of cellulose is a disaccharide, i.e., cellobiose (C_12_H_22_O_11_), rich in –OH groups [128,129]. Materials created using this biopolymer are used in various fields: as engineering materials, in biomedical applications, etc. [130]. The type of cellulose used as reinforcement affects the mechanical properties. Additionally, its mechanical properties, depending on the type of cellulose, determine the cell geometry. It is characterized by good resistance to oxidizing agents and strong bases. However, it is very easily hydrolyzed by acids and water-soluble sugars [131]. It may dissolve under the influence of strong acidic solutions [132]. Biodegradable cellulose can be used as a matrix biomaterial and improves mechanical properties such as a strength and stiffness, acting as a reinforcing agent in the production of green composites [133].

Due to its natural occurrence, the full biodegradability and other excellent properties of cellulose make it one of the most promising fillers used for poly(lactic acid) (PLA) composites. One of the most interesting topics in the literature is the improvement of the compatibility of the created composite between hydrophilic cellulose and hydrophobic PLA. One method to improve the properties of PLA without reducing its biodegradability is to mix it with other biopolymers [134,135]. Cellulose fibers, microfibrillated cellulose, and nanocellulose can affect the properties of PLA, including the regulation of its mechanical, thermal, and antimicrobial properties, degradability, crystallization, and barrier performance [136]. Incorporating the cellulose materials into the PLA matrix significantly reduces costs, making the resulting composite highly competitive with other materials for a variety of applications [137].

Wang et al. prepared an antibacterial poly(lactic acid) (PLA)/cellulose packaging material. Cellulose modified by a silane coupling agent (SCA) was used in this research, which improved the interfacial compatibility between cellulose and PLA. The addition of cinnamaldehyde in the PLA layer provided antibacterial properties. The obtained packaging material effectively inhibited the development of mycelium and spores [138].

de Carvalho Benini et al. reviewed the latest research developments regarding the use of poly(lactic acid) (PLA) and the incorporation of cellulose as a reinforcing agent into this polymer matrix, along with the application of 3D printing technology. Researchers focused their attention on aspects such as the scale and amount of cellulose added to the PLA matrix, the modifications that cellulose surfaces undergo, the incorporation of additives and compatibilizing agents into the PLA–cellulose materials, and the resulting impact of these variables on their properties [139].

Wan et al. prepared new nanocomposites: electrospun stereocomplex PLA fibers (Sc fibers) and PLLA-grafted cellulose nanocrystals (*g*-CNC). Both nanofillers can be used as nucleating agents that will promote the crystallization of PLLA. These works constitute new considerations in the design of various nanofillers, especially for polymer matrices with a low crystallization ability and brittleness [140].

The addition of modified nanocellulose/microcrystalline cellulose or lignin significantly enhanced the mechanical and thermal properties of PLA, and also its crystallization behaviors [141]. PLA/polybutylene succinate (PBS)/cellulose fibril scaffolds exhibited improved proliferation of cells [142]. Cellulose–PLA wound dressings were found to be advantageous in regenerative medicine and also useful in drug delivery systems [143].

#### 3.7.1. Nanofibrillated Cellulose (NFC)

To improve the properties of PLA and increase its application in various fields, it should be modified to include nanomaterials as a reinforcement of the polymer matrix by including, among others, nanofibrillar cellulose (NFC) and nanocellulose (NC). NFC is a nanofiller, which is one of the best-researched materials showing aspects such as renewability, lack of toxicity, and low emissions [144]. Moreover, it is characterized by a low acquisition cost, high strength, and a low thermal expansion [145,146]. The addition of NFC into polymer materials based on PLA can improve the thermal and mechanical properties of the material, causing the material to be successfully used in various fields, including even the biomedical industry [147,148].

Kelly et al. described an aqueous cellulose nanofibril (CNF) grafting polymerization method, which improved the spray-drying properties and strengthening ability of PLA composites. The material is more resistant to stretching. The tests confirmed the better properties of the new CNF/PLA composites [149]. Zhang et al. developed an ecological dispersion of poly(lactic acid) composites reinforced with cellulose nanofibers. The CNF/PLA material for 3D printing was produced using twin-screw extrusion. The CNF modification led to the improved mechanical properties of PLA [150]. Mao et al. studied PLA-based nanocomposite films, reinforced with NFC. They confirmed that the addition of NFC reduced the shear viscosity and shear stress of the nanocomposite suspensions. The reinforced PLA-based nanocomposite films were recommended as a promising material for use in food packaging [151]. Wang et al. also developed biocomposite fibers from cellulose nanofibrils and poly(lactic acid). The CNF/PLA material was prepared by extrusion with of a blend of melted PLA with CNFs as the filler. The results proved that the addition of CNFs improved thermal stability and increased tensile strength. This work provides new possibilities for the use of CNFs in the PLA matrix, creating opportunities in the applications of new materials [152].

#### 3.7.2. Hemicellulose

Hemicellulose has also attracted significant attention in the development of functional polymeric materials. The modifications used are a strategy to achieve the appropriate structure of hemicellulose, giving it the desired properties and improving its compatibility with various matrices. Hemicellulose has unique properties such as environmental friendliness, renewability, and biodegradability [153]. Zhu et al. presented a review of the literature regarding the proportion of hemicellulose and lignin content on the mechanical properties of a PLA composite reinforced with sisal fibers. The use of additives plays an important synergistic role in the effective strengthening of a polymer matrix. Depending on different additives, fibers with different morphologies, compositions, and properties can be obtained. Additionally, by mixing and filling with the polymer matrix, composites with very good properties were obtained [154].

### 3.8. Chitin

**Figure 11:**
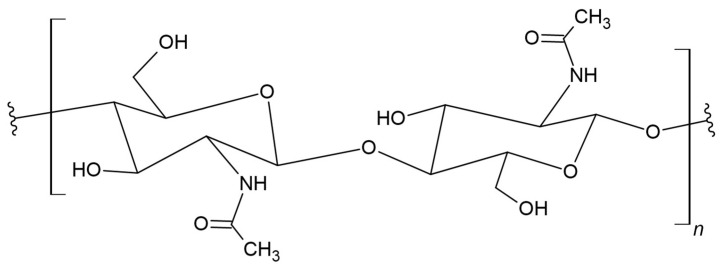
The chemical structure of chitin.

Cellulose and chitin occur in large quantities in nature and are renewable and biodegradable polymers that can be obtained from both animals and plants. Chitin, after cellulose, is one of the most abundant natural polymers and is synthesized by a huge number of living organisms [155,156]. Chitin is the second most readily available polysaccharide, with promising applications in various biomedical fields [157]. Chitin is a building component of the shells of crustaceans and insects. It is also found in the external skeletons of arthropods and in the cell walls of fungi [158,159,160,161]. The chemical structure of chitin is similar to that of cellulose. It consists of N-acetyl-D-glucosamine monomer units that form long polymer chains through β-1,4-glycosidic bonds (Figure 11) [162].

It has chelating properties, forming complexes with other substances. However, its difficulty in dissolution is a serious problem in the development of both processing, which limits its application, and its characteristics. Therefore, it is an underutilized biomass. Recently, with technological progress and better understanding of its physiological and biological properties, it has become a biopolymer widely used in many fields, including the biomedical, pharmaceutical, food, and cosmetic industries [163,164,165]. The structural properties of chitin determine its applications, constituting promising materials for future applications as versatile polysaccharides.

Mansingh et al. presented a composite material intended for packaging edible and health products using existing 3D printing production technology. The addition of chitin to the PLA matrix resulted in a reduction in the strength and stiffness of the material, which was attributed to the reduced interfacial bonding between the reinforcement and the matrix. However, the addition of chitin resulted in increased ductility compared to pure PLA. To sum up, it can contribute to the hardening of the PLA composite. It was also confirmed that as the concentration of chitin in the material increases, the density of the composites increases. Based on the properties of the chitin/PLA blend, it was concluded that the composite material may be useful for applications such as food packaging [166].

Nasrin et al. investigated the possibility of using chitin from available shrimp shell waste into PLA-laminated composites, with the aim of creating novel materials with excellent mechanical and thermal properties for applications in the biomedical sector, such as bone or dental implants [167].

Chitin is very often chemically modified into chitosan, which finds many practical applications (see Section 3.1).

### 3.9. Starch

To make PLA a more favorable option, petroleum-based polymers can be used instead in a wide range of applications, including in the production of other polymers, food packaging, and medical applications; PLA can become an alternative material, which can be mixed with another component that is much more economical [168]. Therefore, starch may be an attractive additive due to its unique properties and low cost, creating materials with the potential for applications in biomedical and environmental fields [169,170,171].

**Figure 12:**
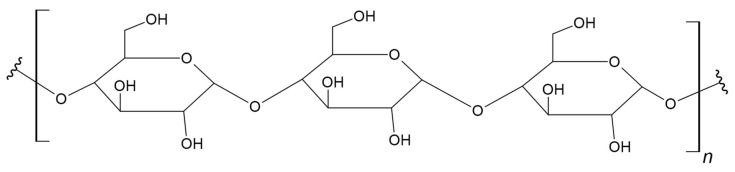
The chemical structure of starch.

Starch is a widespread natural and very important compound found in plants. It consists of two types of polysaccharides, including: from amylose, a linear α-(1→4)-linked glucan, and an α-(1→4)-linked glucan with α-(1→6) branch linkages, called amylopectin (Figure 12) [172,173].

Both combinations form a water-insoluble granule. The ratio of the amount of amylose and amylopectin in starch depends on its biological source, which results in slightly different physical properties of the material [174]. A lot of effort went into designing and developing PLA/starch materials to achieve lower raw material costs and increase their degradability. The interfacial difference between the hydrophilic starch granules and the hydrophobic PLA causes the mixing problem. The incompatibility affects the poor mechanical properties of the resulting PLA and starch mixture [175,176,177,178,179]. The added starch dispersed in the hydrophobic PLA matrix in the PLA/starch blend could be protected from contact with water, while the hydrophobic PLA would form an outer layer to lower the surface tension of the material and thus improve the water resistance of the resulting material [180,181]. However, PLA with a dispersed starch phase produced a weaker and even more brittle material than pure PLA. Many researchers have therefore developed various methods for curing PLA/starch polymer blends to obtain balanced mechanical properties suitable for a wide range of short-term applications [182].

Ávila-Orta et al. developed PLA/thermoplastic starch (TPS) blends with good miscibility and processability, using starch modified by reactive extrusion. The resulting starch-based blend fiber was made possible by using PLA as the main biodegradable polymer. In the blowing process, starch-based nonwovens with interesting properties were obtained. Depending on the amount of starch added, flow properties and fiber diameter reduction, porosity morphology, stiffness, and water affinity can be optimized for specific applications [183].

The combination of PLA and starch biopolymer improved the biodegradation properties and hydrophilicity of the fibers. Donizette Malafatti et al. electrospun a mixture of PLA with starch fibers having good uniformity and particle size below the micrometer scale, being a polymer matrix in the form of nanofibers that can act to release the micronutrient manganese as a model component. Tests of the PLA/starch blend with a content of 20% (*w*/*w*) ensured better affinity of the fiber to water, which is of fundamental importance for the degradation time of the fiber [184].

The addition of the TPS increased compostability, and the material showed a decrease in tensile strength, in Young’s modulus, and in deformation, compared to the PLA matrix after the addition of 30% thermoplastic starch. To improve their mechanical properties, the mixtures were reinforced with bleached kraft hardwood fibers [179]. The addition of TPS effectively improved the mechanical properties of PLA. When the amount of TPS added was 40% by weight, the mechanical properties of the PLA/TPS composites were the best and the elongation at break increased by more than four times. The addition of TPS promoted the crystallization of PLA and reduced the thermal stability of the material. However, during the material processing, its degradation behavior was limited, which had little effect on the performance of the composite [185].

### 3.10. Glukomannanu

Materials intended for contact with food made from natural and biodegradable materials can both reduce environmental pollution and prevent foodborne diseases [171,172,186]. Konjac glucomannan (KGM) is a neutral, water-soluble macromolecular polysaccharide that is natural, biodegradable, non-toxic, and cheap, which influences its wide application. KGM is an important compound that plays various roles in chemical, biological, food science, and medical applications [187,188]. It has high potential as a tissue engineering material based on its excellent biocompatibility, nutritional value, and stability [188].

**Figure 13:**
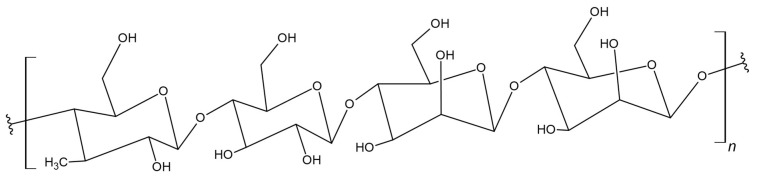
The chemical structure of glukomannanu.

It is composed of hydrophilic glucose and mannoses units. Its main chain and side chain are connected by β-1,4 glycosidic bonds (Figure 13) [189,190]. In connection with the with poor antibacterial activity, low mechanical strength, and difficulties in short-term mass preparation of PLA, KGM-based packaging showed improvements in these properties. Due to the fact that the natural KGM biopolymer is not available for creating microfilms, due to its poor mechanical properties [191], PLA, which has strong mechanical tensile strength, was used to prepare them. It may contribute to the formation of microfilms, meeting the requirements for food packaging materials [192]. Lin et al. developed innovative food packaging using a natural active compound. Glucomannan/poly(lactic acid)/trans-cinnamic acid was used to construct microfilms using microflow spinning technology. The material had good compatibility through hydrogen bonds in microfilms, good swelling ratio, and excellent mechanical properties, thermal stability, and hydrophobicity. Konjac glucomannan was used for the appropriate release of the active substance. To construct active microfilms, antibacterial trans-cinnamic acid (t-CA), a naturally occurring phenolic compound found in various plant sources, was used. The valuable properties of the obtained materials suggest their potential applications in active food packaging [191].

### 3.11. Alginic Acid and Alginates

**Figure 14:**
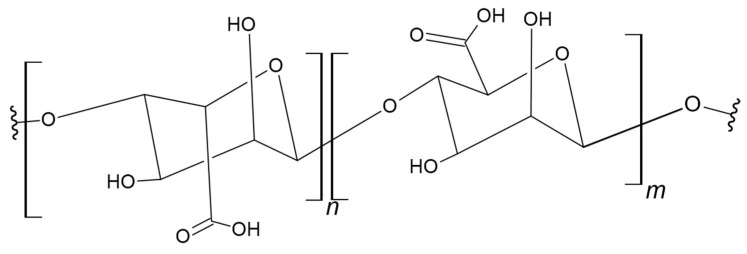
The chemical structure of alginic acid.

Alginic acid (also called algin) and alginates are organic chemical compounds, i.e., hydrophilic polysaccharides, which consist of blocks of alternating residues of β-D-mannuronic acid and α-L-guluronic acid (Figure 14). They are obtained from seaweed or soil bacteria [193,194].

Depending on the source and processing, alginates have a broad molecular weight distribution of 10–1000 kDa [194]. The enzymatic degradation of alginate polymer chains is impossible because the appropriate enzyme is missing [195]. Due to the rapid formation of ionic complexes with divalent cations, e.g., Ca^2+^, alginic acid and alginates have been extensively studied as biomaterials [196,197]. Ibrahim et al. developed a composite, using a blend of alginate with PLA. The resulting diverse structures due to different alginate contents may provide features suitable for various future biomedical applications [198]. Using the centrifugal spinning technique, unique microstructures consisting of PLA microspheres along alginate fibers were formed. Rheological characterization performed showed that the filler (alginate) provided the shear-thinning properties desired for printing and other related applications. This work presented a comprehensive study of biocompatible networks of PLA–alginate microgranules embedded in nano-sized fibers and their potential application in a drug delivery system [198].

In recent years, there has been an increased interest in biodegradable polymers with antibacterial properties for biomedical applications. Kudzin et al. focused on the development of new antimicrobial polylactide/alginate/copper composite materials (PLA–ALG–Cu^2+^). The results obtained showed that this composite can be used as an antimicrobial wound dressing [199]. Promising results inspired the research group of Kudzin et al. to investigate the properties of an antimicrobial and degradable composite material consisting of poly(lactic acid) nonwovens, sodium alginate, and zinc ions. Tests on the PLA/alginate/Zn^2+^ composite confirmed its potential biomedical use as a material with antimicrobial properties [200].

Bîrcă et al. obtained a material intended for the healing of diabetic wounds using alginate hydrogel enriched with Matrigel embedded in poly(lactic acid) (PLA) microspheres containing hydrogen peroxide. The synthesis and characterization of properties of this new material were described. Biological in vivo studies on the wounds of diabetic mice were also performed. The developed composite material accelerated wound healing and promoted angiogenesis in diabetic skin injuries [201]. Noroozi et al. developed a 3D-printed triple periodic minimal surfaces composite scaffold based on poly(lactic acid) and cell-loaded alginate hydrogel. The material showed improved mechanical properties of alginate hydrogel with appropriate pore size. The results showed that the alginate composite scaffold could provide greater cell viability and proliferation [202].

### 3.12. Dextran

**Figure 15:**
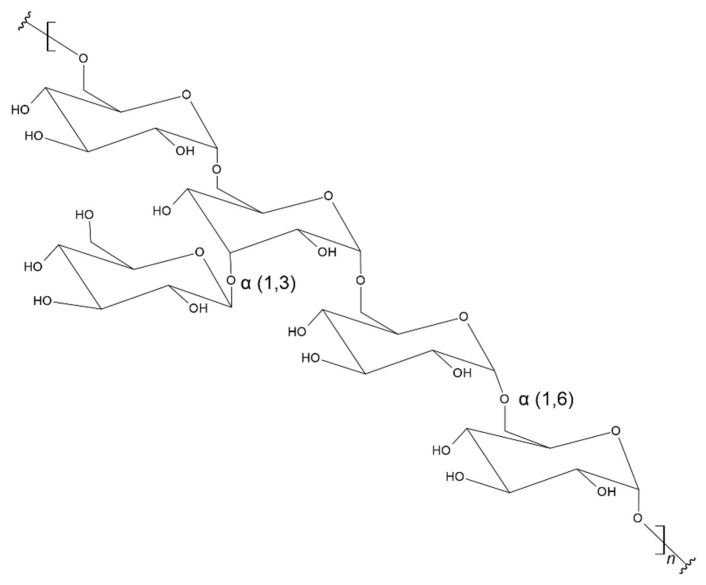
The chemical structure of dextran.

Dextran as a polysaccharide is also not found in human tissues. It is produced by bacteria, mainly from the mucus covering the bacterial cells of *Leuconostoc mesenteroide*. Dextran is found, among others, in chestnut fruits. Dextran is a complex glucan whose structure consists of linear D-glucoses connected by α-(1→6) bonds with possible branches of D-glucoses with α-(1→3) bonds and occasionally at α-(1→4) positions, or α-(1→2) (Figure 15) [203,204,205]. It is characterized by good solubility in water and is easy to functionalize thanks to the reactive hydroxyl groups in the structure [206,207]. It is of interest as a biodegradable and biocompatible material [208]. Its degradation occurs by natural enzymatic cleavage of bonds by the action of dextran-1,6-glucosidase, which can be found in the liver, lungs, kidneys, spleen, brain, and muscle tissue, and degradation also occurs by dextranases produced by bacteria in the large intestine [209,210]. Studies have also confirmed that dextran is resistant to protein adsorption, and the relatively low cost, availability of dextran, as well as its hydroxyl group functionality for chemical modification have increased the interest in its use in biomaterials [211,212,213].

PLA and dextran have been widely investigated for biomedical applications. Due to the hydrophobic nature of PLA, which makes this polymer much less desirable in the case of cell adhesion. The polysaccharide dextran is highly hydrophilic and is not very useful in tissue engineering because it dissolves easily in water. Zhang et al. prepared biodegradable, fiberized PLA scaffolds with dextran, which influenced the hydrophilic nature of the nanofiber. Nanofibers with different PLA-*g*-dextran compositions (10–88% dextran) were crosslinked by grafting dextran onto PLA. The results showed that nanofibrous scaffolds, depending on the dextran content, can significantly increase adhesion and also influence cell differentiation and biological activity [214]. J. Raynaud et al. synthesized poly(lactide)-grafted dextran copolymers (Dex-*g*-PLA). Depending on the content of dextrin in PLA, these copolymers showed solubility in water or organic solvents, and they were also able to stabilize direct or reverse emulsions [215].

A novel concept of stereocomplex formation, i.e., self-assembly of enantiomeric lactic acid oligomers conjugated with dextran, obtaining dex-(L)lactate and dex-(D)lactate, respectively, was described by the group of de Jong [216]. Zhang et al. used UV photopolymerization to obtain a new class of biodegradable hydrogels, which consisted of poly(D,L-lactic acid) (PDLLA) and hydrophilic dextran segments with a polymer network structure. Hydrophobic segments of PDLLA and hydrophilic dextran intermixed in network hydrogels that were characterized by a wide range of hydrophilicity to hydrophobicity [217].

Hydrogels can contain large amounts of water, which makes them a promising material, among others, for protein delivery. Hennink et al. developed a biodegradable dextran hydrogel based on physical interactions, showing the potential for controlled delivery of pharmaceutically active proteins. Scientists engineered gel formation by eliminating organic solvents and/or cross-linking agents by mixing aqueous solutions of dextran(*L*)-lactate and dextran(*D*)-lactate. This system can be used as a controlled release matrix for pharmaceutically active proteins [218].

Xiao et al. elaborated on multifunctional hydrogels combining the thermoresponsive and biodegradable properties of polymeric materials, which consisted of *N*-isopropylacrylamide (NIPAAM) as a thermoresponsive component, PLA as a hydrolytically degradable and hydrophobic component, and dextran as an enzymatically degradable component with hydrophilic properties. Given their wide range of properties, the resulting hydrogels have great potential for applications in the biomedical field, including drug delivery and tissue engineering [219]. Huang et al. developed, characterized, and tested the newly obtained hydrogel material, which consisted of NIPAAM, poly(D,L-lactic acid) and dextran segments. The obtained material as a cartilage tissue engineering scaffold for in vitro chondrocyte culture has been shown to be effective for seeding chondrocytes that have retained their phenotype [220]. Dextran is a widely available biocompatible material that is easy to crosslink and functionalize. It works by repelling unwanted adsorption of cells and proteins. Biofunctional dextran-based materials address some of the most important challenges in 3D cell culture, thereby ensuring precise definition and specificity of scaffolds [221,222].

Dai et al. prepared a new material from poly(D,L-lactic acid) (PDLLA) and dextran. The amorphous structure had the advantage of rapid degradation properties. Moreover, the PDLLA-dextran nanodrug showed an excellent structural stability and excellent biodegradability in multi-drug combination treatment for Alzheimer’s disease [223].

### 3.13. Hyaluronan

**Figure 16:**
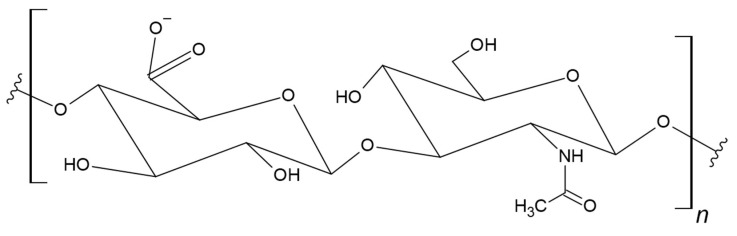
The chemical structure of hyaluronan.

Hyaluronan, also known as hyaluronic acid (HA), is a linear hydrophobic glycosaminoglycan. This biopolymer consists of alternating units of D-glucuronic acid and *N*-acetyl-D-glucosamine connected by β(1→4) and β(1→3) glycosidic bonds (Figure 16) [224,225].

This acid is a component of the intercellular matrix of the dermis and is also the main component of synovial fluid [226,227]. Depending on its occurrence, hyaluronic acid has a molecular weight of up to 10,000 kDa [228]. To obtain hyaluronic acid, it can be extracted from living cells; however, due to the reduced risk of cross-species viruses, infections, and contamination, HA is mainly produced by microbial fermentation [229,230]. Research on HA confirms its impact on cell–cell and cell–substrate adhesion, cell proliferation, and migration, and helps organize proteoglycans and bind collagen and fibrin. HA has also been confirmed to support angiogenesis and wound healing [231]. These properties mean that it has a wide range of medical applications [232,233,234,235,236,237,238,239]. Rapid enzymatic degradation in the body has limited the usefulness of HA as a long-term implant biomaterial [240]. To control and slow degradation, synthetic polymers are often combined with HA. This combination can increase the mechanical strength of the materials [241].

In order to improve solubility and bioavailability, a preparation based on self-assembled nanoparticles of hyaluronic acid poly(lactic-*co*-glycolic acid) (PLGA) was prepared for local use in oral cancer cells (TR146 cell line). The developed nanocarrier loaded with the Ru(II) complex may be potentially effective in the treatment of oral cancer [242]. Yun et al. obtained PLA scaffolds, by using 3D printing, that were biocompatible and integrated well with a bone defect. The use of hyaluronic acid in 3D-printed PLA facilitated the formation of a new bone [243]. Roca et al. investigated a novel, multi-modular nerve conduit based on a microfibrillar structure of poly(lactic acid) (PLA) placed inside several co-linear HA conduits for the treatment of large nerve injuries. The PLA microfibers used provide a topographic cue to guide axonal growth, and the HA played the role of epineurium and retain pre-seeded support cells. The multi-modular approach contributed to the regeneration of large nerve defects, which opened new possibilities for surgical solutions in this field [244]. Niu et al. also functionalized PLA tubular microfibers with hyaluronic acids. The HA/PLA microfibers were created by electrospinning and showed no detectable signs of hemolysis and coagulation. Moreover, the obtained micromaterials promoted vascular endothelial cells (ECs) proliferation and phenotypic expression. Studies confirmed that HA/PLA enhanced luminal pre-endothelialization of vascular ECs in vitro [245].

PLA-HA composites containing 20 wt.% of HA (particle size—50 μm), obtained by FDM technology (Fused Deposition Modelling), presented the best bone integration combined with improved mechanical properties (excellent elastic modulus of 10.12 MPa and compressive strength of 31.18 MPa) [246].

### 3.14. Chondroitin Sulfate

**Figure 17:**
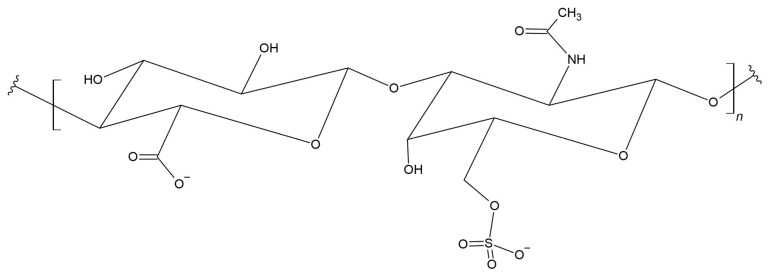
The chemical structure of chondroitin sulfate.

Chondroitin sulfate (CS) is an organic chemical compound from the group of glycosaminoglycans, a mucopolysaccharide consisting of a linear chain of alternating glucuronic acid and N-acetylgalactosamine residues (Figure 17) [247,248]. CS was originally isolated long before its structure was characterized [249].

Chondroitin sulfate is a type of a linear polysaccharide that forms proteoglycans by covalently linking to proteins [250]. Chondroitin sulfate proteoglycans (CSPGs) occur on the surface of cells and in connective tissues, interacting with many proteins that are involved in various pathophysiological processes. CSPGs are often considered in relation to the role in which a given proteoglycan regulates a specific role in cell physiology. CSPGs are important components of connective tissue that fine-tune a wide range of cellular processes, including: growth factor signaling, neuronal development, and inflammation [251]. The key factor is the number of disaccharide units forming the chondroitin sulfate polymer, which influences biological and pharmacological activity [252].

Zhang et al. modified the PLA surface by introducing carboxyl functional groups in photooxidative modification and then using amino terminated polyoxyethylene NH_2_-PEG-NH_2_ as an intermediate to graft chondroitin sulfate (CS) onto PLA. Scientists confirmed that endothelial cells produced using the CS method adhere better to PLA and were resistant to platelet adhesion [253]. Fajardo et al. described the properties of the synthesis and self-assembly of chondroitin sulfate-*b*-poly(lactic acid) (CS-*b*-PLA)_n_ in water. The diblock copolymer self-assembled in water, forming spherical micelles. Moreover, it did not show any toxicity. The obtained material can potentially be used as a biocompatible nanocarrier for the delivery of anticancer drugs [254].

### 3.15. Heparin

**Figure 18:**
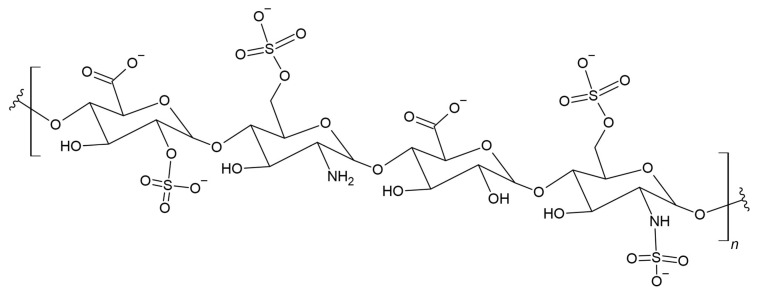
Chemical structure of heparin.

PLA is a polymer often used in biomedical applications, including: in the fields of tissue engineering, nanotechnology, gene therapy, or drug delivery systems, it can act as implants due to its biocompatibility and biodegradability [255,256]. Cell compatibility and thrombus formation are still a serious problem in the use of biodegradable PLA, and research on the properties and effects of polymeric materials in contact with tissues was not very often studied [257,258,259]. Heparin has many biological activities that are related to interactions between proteins. Heparin (Figure 18) and heparan sulfate regulate biological processes through interactions with a large number of proteins [260]. Heparin is very often used as an anticoagulant and antithrombotic drug [261]. Newer studies focused on incorporating it into biomaterials through dispersion [262,263].

Interest in the interactions of heparin with proteins that perform important functions during physiological processes resulted in its use not only as an antithrombotic agent [264]. The bioactivity of heparin bound to degradable polymers is expected to be promising materials for minimizing or preventing thrombus formation where blood contact occurs in degradable polymer applications, as well as regulating cell growth and differentiation in cell-matrix interactions used in tissue engineering [265]. Sheng et al. performed the latest literature review on the use of heparin in biomaterials, including PLA [266].

Go et al. developed a novel material, which was prepared by coupling heparin (Hep) to the star-shaped PLA (sPLA) using carbonyldiimidazole (CDI) chemistry. It is an improved polymer material that is degradable and compatible with biological material, among others, with cells and blood due to the immobilization of heparin on the end group of multivalent sPLA. This combination resulted in the creation of a hydrophilic environment on the surface of the material, and in lower protein adsorption and platelet adhesion. The addition of heparin increased cell activity. The suitability of sPLA-Hep in biomedical applications as cell-compatible biodegradable materials for implantable medical devices and tissue engineering was confirmed [267].

## 4. Lipid-Modified Poly(lactide) Scaffolds

**Figure 19:**
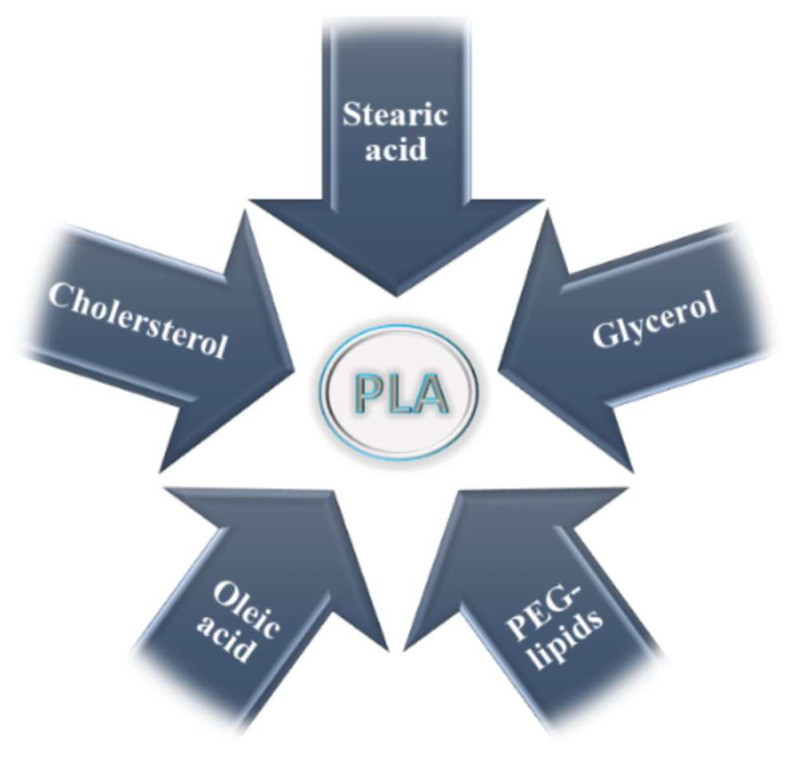
A list of lipids and other compounds combined with PLA.

Although the term “lipids” is sometimes used as a synonym for fats, they are actually a subgroup of fats, the triacylglycerols. Lipids include fats, waxes, sterols (including cholesterol), fat-soluble vitamins (A, D, E, K), monoacylglycerols, diacylglycerols, phospholipids, and many other substances. Lipids are small hydrophobic or amphiphilic molecules that form vesicles, liposomes, or membranes in an aqueous environment. Biological lipids can be divided into eight groups: fatty acids, glycerolipids, glycerophospholipids, sphingolipids, glycolipids, and polyketides (derivatives of condensation of ketoacyl subunits), sterols, and prenyl lipids (condensation products of isoprenoid subunits). The main biological functions of lipids are energy storage, the formation of biological membranes, and participation in signal transduction [see in Wikipedia]. Lipids have long carbon chains. Fatty acids were first isolated and identified by Michel-Eugène Chevreul in 1823 [268]. Saturated and unsaturated fatty acids can be distinguished, which may contain one or more double bonds between carbon atoms, and at the end of the chain a functional carboxyl group exists [269]. Lipids, cholesterol, fatty acids, and glycerol were used for modification of PLA properties (Figure 19).

Fatty acids have a wide range of applications in technology and many areas, including cosmetic, pharmaceutical, biomedical industry, etc., and are also used in the plastics and rubber industry. These acids can perform a variety of functions, ranging from surfactants, softeners, dispersants, through to activators and surface modifiers [270].

### 4.1. Stearic Acid

**Figure 20:**
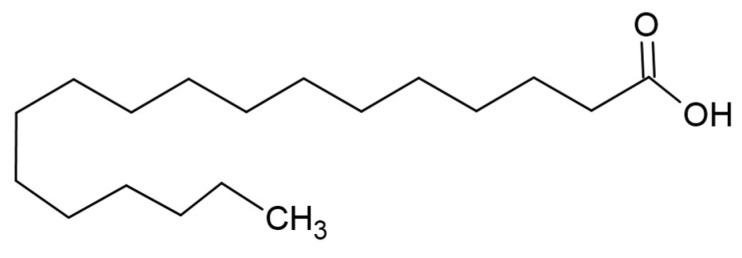
The chemical structure of stearic acid.

Stearic acid (Figure 20) can be obtained by hydrolyzing animal fats or hydrogenated cottonseed or vegetable oil. The commercial grade stearic acid is a mixture of stearic acid with palmitic and myristic acids [271].

Andrade et al. prepared and characterized biocomposite poly(lactic acid) (PLA) fibers combined with a nano-hydroxyapatite (n-HA) filler, coated with stearic acid (SA) as a surfactant to improve rheological properties. The coating reduced the brittleness of the PLA/n-HA fibers. The developed scaffolds had the necessary mechanical, thermal, and cytotoxic properties for applications in bone tissue engineering [272].

### 4.2. Glycerol

Increasing attention is being paid to the study of renewable and biodegradable polymer plastics due to the increase in pollution resulting from landfills of non-biodegradable plastics [273]. Therefore, PLA is considered as a promising material to replace non-biodegradable plastics. To increase the use of PLA in everyday life, it is necessary to overcome the cost of producing this polymer by mixing PLA with other polymers or fillers, which will also affect its degradation rate [93,274]. The world needs balance in economic, social, and environmental development. Biofuels are the basis; they will eventually replace conventional fossil fuels and make society sustainable [275].

**Figure 21:**
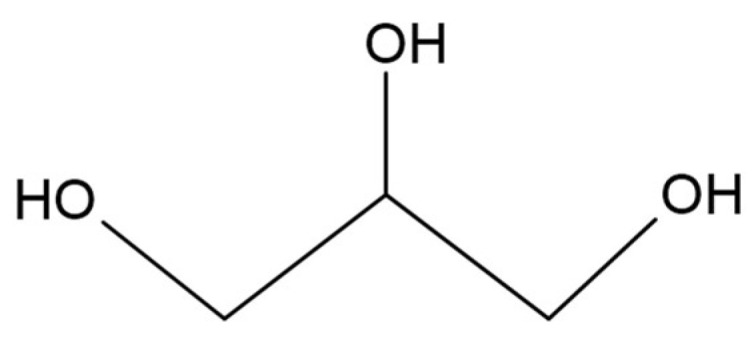
The chemical structure of glycerol.

As a result of the transesterification reaction during biodiesel production, a crude glycerol is produced as a by-product. It is known as propane-1,2,3-triol (Figure 21), which is used in many pharmaceutical, food and personal hygiene everyday products, having antimicrobial and antiviral properties [276,277,278]. Satriyatama et al. investigated the addition of glycerol in PLA/wheat bran blends, which improved the mechanical properties of the obtained material. This research focused on developing polymer products for the packaging industry or for use in the biomedical sector while being environmentally friendly [279]. Lv et al. studied the effect of glycerol on the photodegradation process of starch/wood flour/PLA composites. It was confirmed that glycerol has a stabilizing effect on the durability of composites against UV radiation [280].

Li and Huneault investigated the use of glycerol as a plasticizer for thermoplastic starch (TPS) in TPS/PLA blends. The analysis of morphological, rheological, and mechanical properties showed that glycerol is susceptible to transfer from the thermoplastic starch phase to the PLA polymer phase. Glycerol migration during the melting phase led to a higher viscosity ratio between the dispersed TPS phase and the PLA matrix, leading to thicker morphologies of the resulting blend. In the solid state, however, the transfer of glycerol to the polymer matrix led to lower tensile strength and modulus, but to a higher crystallization rate when heated due to higher chain mobility [281].

Valerio et al. prepared polyesters based on, among others, glycerol to obtain a bioproduct suitable for hardening PLA using melt mixing technology. In order to find the optimal synthesis conditions leading to the highest increase in the strength of PLA, the molar ratios of the reactants and the temperature of the synthesis were examined. Therefore, it was found that a 1:1 molar ratio of glycerol/succinic acid (SA) reagents increased the effectiveness of poly(glycerol succinate) (PGS) as a toughening agent for PLA. Alternatively, maleic anhydride (MA) was used a comonomer for the synthesis of the partial replacement of SA, giving poly(glycerol succinate-*co*-maleate), PGSMA, which was advantageous for making copolymers suitable for reactive extrusion (REX), mediated by free radical initiators. The final REX PLA/PGSMA material was characterized by the tensile strength (TS), and the hardness of the mixtures increased by lowering the temperature. It was found that the optimal conditions for the synthesis of PGSMA, were 1:0.5:0.5 mol of glycerol/SA/MA, respectively, leading to the highest increase in strength of PLA/PGSMA blends. The TS of the REX PLA/PGSMA blends was improved by 392% in comparison with that of neat PLA, due to the simultaneous cross-linking of PGSMA within the PLA matrix, and the in situ formation of PLA-*g*-PGSMA graft copolymers acting as interfacial compatibilizers [282].

### 4.3. PEG-Lipids

Poly(ethylene glycol (PEG) lipid (PEGylated lipid) is a class of PEG derivatives that play important functions in formulation strategies. They are characterized by a PEG skeleton with a lipid end. They are the interesting class of compounds because they are used in the commercial production and distribution of the most successful vaccines [283]. Duncanson et al. obtained polymer particles consisting of PLA with built-in PEG-lipids, the research of which is the basis for developing these structures with interesting ligands, obtaining new properties [284].

### 4.4. Oleic Acid

**Figure 22:**
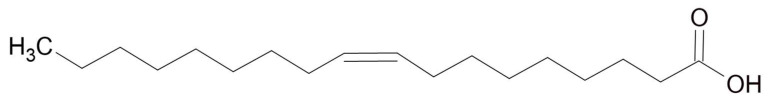
The chemical structure of oleic acid.

Oleic acid (Figure 22) is a fatty acid containing carbon atom and a cis-double bond in the 9-carbon position. Commonly designated as *omega*-*9* fatty acid, which is widely distributed, among others, in animal, plant, tree nut, marine, and algae lipids [285].

Oleic acid is widely used as a plasticizer and functional modifier to obtain better dispersions of hydrophilic nanoparticles (NPs) in hydrophobic media and increase barrier properties. Baek et al. determined the impact of a surface modification of TiO_2_ with oleic acid on improving the dispersion of NPs in the PLA matrix. Surface-modified TiO_2_ by oleic acid improved the dispersion of NPs in PLA matrices. The addition of oleic acid improved the barrier properties for oxygen and water vapor. Additionally, an increased elasticity and reduced brittleness of the material thus created was observed [286]. Yin et al. prepared a PLA composite with nickel ferrite nanoparticles with an oleic acid surface coating, which were encapsulated in PLA microspheres. A slight decrease in the glass transition temperature and cytotoxicity was observed in the obtained composite materials [287].

### 4.5. Cholesterol

**Figure 23:**
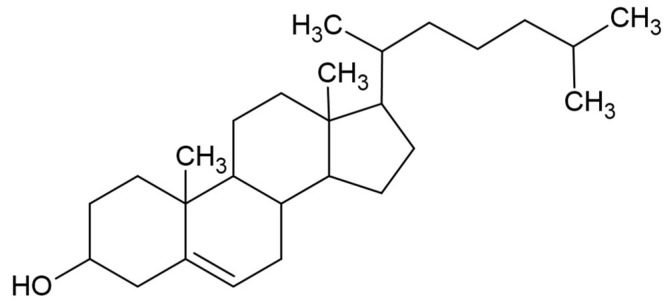
The chemical structure of cholesterol.

Biological barriers provide protection against harmful substances or pathogens, while also being a challenge to overcome in drug delivery. Therefore, new strategies are being developed [288]. One of the structural elements of cell membranes is cholesterol. This ingredient is a building block in the synthesis of vitamin D and bile acids and various steroid hormones [289]. Moreover, it plays a key role in regulating processes affecting cell functions [290]. Among the biocompatibility of lipids, including phospholipids, cholesterol is biocompatible and biodegradable, and is also characterized by a low toxicity [291]. Cholesterol is composed of a tetracyclic fused ring skeleton, a double bond at carbon 5 and 6, an isooctyl hydrocarbon side chain at carbon 17, and a hydroxyl group at carbon 3 (Figure 23) [292].

The hydroxyl group imparts an amphiphilic character by orienting the cholesterol molecule in membranes. Additionally, the hydroxyl moiety may mediate the formation of hydrogen bonds between cholesterol and water and probably other lipid compounds that are components of cell membranes. The cholesterol molecule is flat and rigid because it exists in the trans conformation except for the flexible isooctyl side chain [293].

Kumari et al. identified a new micellar drug delivery system based on cholesterol-modified mPEG-PLA micelles (mPEG-PLA-Ch). The created material had improved core hydrophobicity in the micelles, which effectively encapsulated and delivered the drug in various cancer cell lines in vitro and into the tumor in vivo, being a potential material in a promising anticancer therapy [294]. Yu et al. obtained PLA–cholesterol oligomers by mass polymerization. The tests confirmed efficient and controllable polymerization. Moreover, the PLA–cholesterol oligomers did not show any toxicity towards osteoblast cells and may have potential applications in bone tissue engineering [295].

## 5. Polylactide Scaffolds Modified with Proteins

Polylactide (PLA) is hydrophobic, which means it prevents cell adhesion to the polymer surface [296]. By modifying the surface with extracellular matrix proteins, the hydrophilicity of PLA-based scaffolds can be increased [297]. Proteins, together with nucleic acids, lipids, and polysaccharides, constitute the main molecules necessary for life. All the above molecules are covalent and have a carbon-based structure. Proteins are polymers usually containing sequences of amino acids linked together by peptide bonds [133]. Amino acids containing a central α carbon atom (Cα), which is connected to an amino group (–NH_2_) and a carboxylic acid groups (–COOH), which are reactive groups for the formation of peptide bonds [298]. The α-amino (–NH_2_) and α-carboxyl (–COOH) groups of amino acids are essential components for the synthesis of peptides in living organisms. Apart from glycine, amino acids are chiral molecules that come in two forms, L- and D-enantiomers. Even though they have identical chemical and physical properties, but only D-chirality amino acids are used by organisms [299].

**Figure 24:**
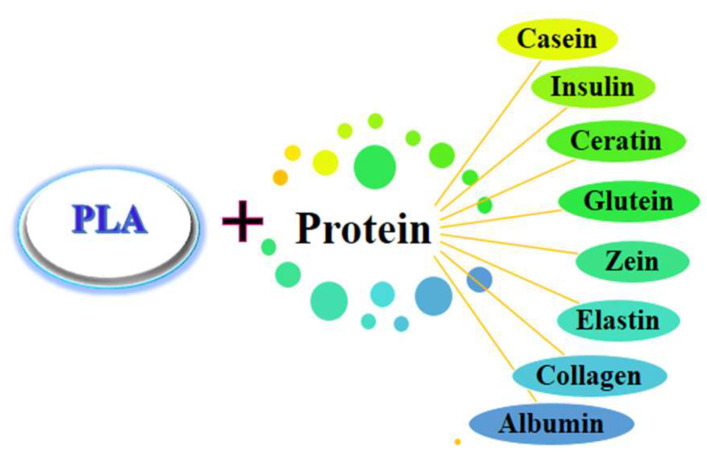
The list of proteins combined with PLA.

Proteins (Figure 24) are extremely attractive molecules as candidates for high-performance bionanocomposites due to their wide advantages, including: easy availability, biodegradability, biocompatibility, and also due to reactive sites in the protein structure enabling new functions to be obtained [300]. These features can be exploited by blending with polymer material, which can significantly improve the structural properties of new PLA-based protein materials. Proteins make a good raw material because they have the advantages of biopolymers along with the advantages of absorbability and low toxicity of final degradation products [301].

### 5.1. Albumin

Albumin is an interesting biomolecule that is an attractive and versatile macromolecular carrier. It is characterized by no toxicity, is non-immunogenic, and is biodegradable. Metabolized in vivo, it produces harmless degradation products. It is easy to purify and soluble in water, which makes it an ideal candidate for the preparation of nanoparticles (NPs) that are of great interest due to their high ability to bind various drugs and are well tolerated without causing serious side effects [297,302]. The albumin-based nanoparticle carrier system is an attractive strategy due to the particle matrix and its various binding sites present in the albumin molecule [303]. Albumin-based NPs could enable electrostatic adsorption of positively or negatively charged molecules without the need for other compounds, because the defined primary structure of albumin has a high content of charged amino acids [304]. Albumin, a globular protein, is commonly found in egg white, milk, plants and blood plasma. It is necessary because it performs many important functions, including those related to the regulation of oncotic pressure, regulation of blood pH, and binding and transportation of bioactive molecules, including amino acids, proteins, peptides, fatty acids, drugs, nutrients, and metal ions, etc. The properties of albumin make it an excellent candidate for applications in the biotechnological and medical fields [305]. Albumin is a simple protein, composed of one chain containing 585 amino acids with a relatively low molecular weight (~66 kDa) and an isoelectric point 5.9 [306].

Well-known polymers such as human serum albumin and poly-D,L-lactic acid are often used in medicine for various purposes, such as the delivery of certain drugs. Buketov et al. described methods for obtaining nanomaterials based on human serum albumin and poly-D,L-lactic acid, which were successfully synthesized by desolvation and nanoprecipitation methods with satisfactory physicochemical properties. The resulting nanomaterials can be used to load them with various drugs [307].

Nyanhongo et al. investigated the possibility of increasing biocompatibility and hydrophilicity and focused on the antioxidant properties of PLA membranes through chemical and enzymatic grafting of thermally denatured human serum albumin. Studies demonstrated that PLA membranes can be activated both chemically and enzymatically, leading to the formation of functional groups that can be further reacted with a heterobifunctional cross-linker, enabling successful human albumin transplantation. Surface modification increased hydrophilicity, radical scavenging capacity of PLA membranes, cell viability, as well as proliferation of osteoblasts and MC-3T3-E1 cells. The hydrophilic groups of albumin promote interactions between cells and PLA membranes, resulting in increased biocompatibility [308].

Verrecchia et al. prepared poly(lactic acid/albumin) “PLA/HSA” nanoparticles (NPs) by solvent emulsification-evaporation and microfluidization. These NPs are particularly interesting because they are fully biodegradable and well-tolerated colloidal suspensions. In vivo studies were conducted in which these NPs were administered daily, i.e., injected into rats, but any visible side effects were noticed. However, the time necessary to remove PLA/albumin NPs from the plasma was very short (approx. 90% were eliminated from the bloodstream within 5 min). In this connection, a new biodegradable hydrophobic diblock copolymer of poly(D,L-lactide)-*b*-poly(ethylene glycol) (PLA-PEG) was designed, where the PEG layer covering the surface of the nanoparticle increased the half-life of the colloidal carrier in the plasma. The half-life of PLA-PEG NPs was significantly extended and was approximately 6 h instead of several minutes as in the case of PLA/albumin-coated NPs [309].

### 5.2. Collagen

**Figure 25:**
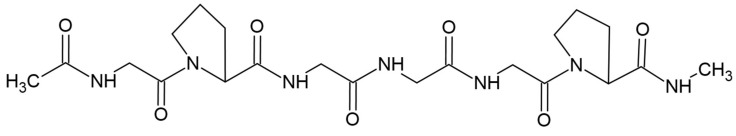
The chemical structure of collagen.

Collagen (Figure 25) can be obtained from several sources. However, the properties of collagen-based materials, i.e., quality and price, are influenced by both the source of collagen and the method of its purification and further processing [310]. It is typically obtained by extraction from natural sources, for example several animal tissues, or by recombinant protein production systems [311].

The incorporation of natural biomacromolecules into PLA structure through graft modification is one approach to improve the biocompatibility of PLA. Collagen is an important extracellular matrix in many tissues with antigenic properties. Due to its biocompatibility and biological properties, such as biodegradability, it makes collagen suitable for cell adhesion and cell proliferation [312]. Currently, the use of collagen as a biomaterial is experiencing great interest. Its biotechnological applications focus on the delivery of proteins capable of stimulating cellular response or cellular growth. Therefore, basic knowledge of collagen biochemistry and processing technology is necessary to properly apply collagen and obtain the desired physicochemical properties of biomaterials [313,314]. Despite the many advantages of PLA, surface modification is still required to further improve its compatibility, because the polymer has hydrophobic properties and has no cell binding site [315,316].

Collagen is a protein that is most common in tissues such as tendons, skin, connective tissue, bones, and cartilage. Collagen plays a very important role in controlling cell adhesion and migration and tissue repair, and also provides mechanical support in tissues [317]. Its biological properties mean that it is used not only in cosmetic applications. It is distinguished by biocompatibility, lack of toxic effect, cell affinity and weak antigenicity, biodegradability, and structural integrity. The above properties have encouraged the use of collagen in biomedical and pharmaceutical applications [318].

Dunn et al. developed collagen fiber composites in a poly(lactic acid) (PLA) matrix, which were characterized by better mechanical properties, i.e., tensile strength and elastic modulus. The research results indicated the potential use of the developed composite in medical applications [319]. There is still a need for new, innovative bone substitutes. Ritz et al. used a 3D printing method using poly(lactide), which was appropriately coated or filled with collagen. These tests confirmed the biocompatibility of PLA and showed that endotoxin contamination was clearly below the FDA (Food and Drug Administration) limit. The collagen-loaded PLA material promoted cell growth, demonstrating the potential of desktop scaffolds in medical applications, including bone tissue engineering [320].

Haaparanta et al. developed three-dimensional (3D) porous collagen/polylactide (PLA)-based scaffolds for the repair of articular cartilage defects. Innovative hybrid materials were produced by combining PLA and freeze-dried natural ingredients. PLA provided mechanical strength, and collagen imitated the natural environment of chondrocyte cartilage. The collagen material showed good cell penetration into the scaffold. The results showed that the tested collagen/PLA material may be a promising scaffold for cartilage tissue engineering [321]. With the aim of developing new biomaterials to support the regeneration of damaged bone, Dyzio et al. described a new mineralized collagen-PLA composite. The results of conducted research suggested that the inclusion of a PLA reinforcing frame did not negatively affected the osteoinductive nature of the mineralized collagen scaffold. The prepared material demonstrated a mechanical strength, bioactivity, and shape adaptation of biomaterials for bone regeneration [322]. To exploit the mechanical strength of PLA and the bioactivity of collagen, Xie et al. designed a collagen/poly(lactic acid) (COLL/PLA) hybrid yarn. Using textile weaving technology, this material was used to create a tissue engineering scaffold. Collagen significantly improved the proliferation of tendon-derived cells. The biological activity of collagen and the mechanical properties of PLA, creating a COLL/PLA hybrid scaffold, make it potentially useful in biomedical applications [323].

### 5.3. Elastin

Elastin is a protein found in connective tissue, providing elasticity and tensile strength to many other tissues and organs, including: skin, lungs, elastic ligaments, blood vessels, etc. These proteins are characterized by a self-organizing structure creating elastic fibers [324]. According to obtained research results, elastin expression was regulated by the positive or negative regulatory effects of various molecules [325].

Elastin is an extremely insoluble protein due to the extensive cross-linking at lysine residues. A chemical structure of elastin is quite complex. Similarly to hydroxyproline-rich collagen, elastin from higher vertebrates, including humans, contains over 30% glycine (Gly) and approximately 75% of its entire sequence is built up of four non-polar, hydrophobic amino acids: glycine, valine, alanine, and proline (Gly, Val, Ala, Pro), including approximately one-ninth proline. Thus, elastin is one of the most hydrophobic proteins known [326,327,328].

**Figure 26:**
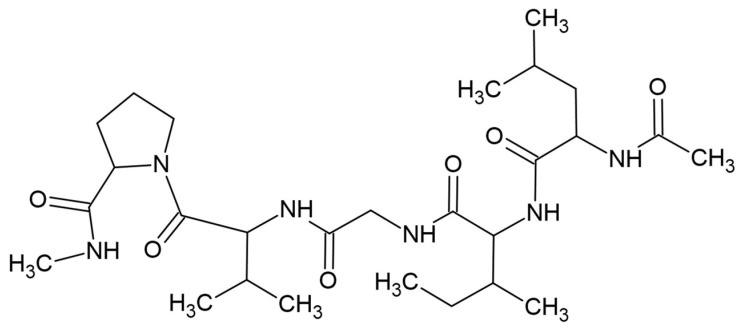
The chemical structure of elastin.

The large amount of hydrophobic amino acids contained in the structure of elastin (Figure 26) makes it chemically resistant and a very durable protein. Fibers of elastin are mostly elastic and are created by the hierarchical combination of its monomer, tropoelastin, which is a non-cross-linked form of elastin and is a key component of elastic fibers [329,330,331].

Due to the potential of natural polymers to generate an undesirable immune response with human tissues, synthetic polymers are more preferred in biomedical applications. However, they are often insufficient materials due to their disadvantages, including their low surface energy. Therefore, hybrid composite materials are often developed, which combine a biological molecule with a polymer [332]. Such composites are also made of elastin and PLA. Tesfaye et al. investigated the effect of a modified elastin-collagen matrix (m-ELA-COLL) on the properties of PLA. The modified material (PLA/m-ELA-COLL) showed an improvement in the degree of crystallinity and increased tensile strength. The research results confirmed that the obtained bioproduct can potentially be successfully used in the packaging industry [333]. The research group of Castillo-Ortega et al., using the electrospinning technique, prepared a material consisting of poly(lactic acid) (PLA), elastin and gelatin fibers, containing clindamycin, which could potentially be used as wound dressings. The fiber exhibited a uniform morphology with hydrophilic properties, thus improving cellular adhesion to the material. The fibers loaded with clindamycin showed antimicrobial activity, which allows the use of the developed material in the treatment of skin wounds [334].

### 5.4. Zein

A chemical structure of zein is also complex. It is composed of many proteins, similarly to gluten, and consists of glutamic acid, leucine, alanine and proline structures. It is found in corn and is recognized by the FDA as one of the safest biological materials [335]. It is characterized by low immunogenicity, is biodegradable, biocompatible and is not harmful to the body when consumed. According to the *α*-, *β*-, *γ*- or *δ*- classification, the main fractions are α- and β-zeins [336].

Zein is a biopolymer that is used to produce nanofibers because it is characterized by strength, non-toxicity, low cost, compatibility, and hydrophobicity. However, electrospun zein nanofibers dissolve quickly and have poor mechanical properties [337,338]. Many non-polar amino acid residues, as well as a deficiency of both acidic and basic amino acids, are responsible for the dissolving properties of zein. Zein is soluble in alcohol, in alkaline solutions, anionic detergents and in solutions with a high concentration of urea. But it is characterized by a lack of solubility in water [339]. Thanks to the possibility of modifying its structure, zein can have various types of micro/nanostructures and has a wide range of applications, including as drug carriers, scaffolds, and also acts as a coating in the food and pharmaceutical industries, because it is characterized by good film-forming properties and a gas barrier [340,341]. Zein-based films can potentially serve as replacement packaging for plastics. However, its barrier properties, elasticity and mechanical properties should be improved by using appropriate additives [342,343,344]. Chen et al. proposed a porous poly(lactic acid) coating on zein films to improve their function using cold plasma (CP) pretreatment. The obtained modified coating films showed a high barrier to UV radiation and excellent biodegradable properties. The strategy used to apply a porous PLA coating on zein foils allowed for improvement of the properties of zein and also extended the scope of application of this material [345].

Altan et al. developed zein-PLA-based composites by incorporating carvacrol at different concentrations via electrospinning. The resulting fibrous composite layers exhibited sustained diffusion-controlled release. This research confirmed its usefulness in food packaging applications to extend shelf life [346].

### 5.5. Fibrinogen

Mixing polymers allows the production of new materials with characteristic structural, mechanical, and biochemical properties. Recent studies have shown that blending natural and synthetic polymers can improve mechanical stability because natural materials are often weaker than synthetic ones [347]. Moreover, mixing synthetic materials with bioactive proteins can impart biological functionality.

Fibrinogen plays important functions in many physiological processes in the body. It is a soluble glycoprotein found in plasma with a high molecular weight of 340 kDa. It is composed of three pairs of non-identical polypeptide chains (*alpha*, *beta*, and *gamma* chains) connected by disulfide bonds [348]. Fibrinogen is converted into fibrin, where it provides a mechanical and structural scaffold for blood clots at the site of blood vessel damage and promotes hemostasis [349]. Fibrin fibers have excellent interactions with cells by promoting cell adhesion, differentiation, and proliferation. They can imitate the natural extracellular matrix and are characterized by high biocompatibility and biodegradability. Despite these attractive features, the interest and attention of researchers in this material is lower compared to other natural polymers, such as collagen or chitosan [350]. Gugutkow et al. developed nanofibers based on a combination of fibrinogen and poly(lactic acid) (FBG-PLA). The material combined the good mechanical properties of PLA with the excellent cell recognition properties of native FBG. These studies showed that electrospun fibrinogen–PLA nanofibers can be used in a vascular tissue engineering [351].

### 5.6. Glutein

Glutein has a complex nature and composition as wells. It shows high allelic polymorphism coding for two kinds of proteins: gliadins and glutenins. The ratio of glutenins to gliadins and the interactions of these structures influence the rheological and functional properties of gluten. Both fractions are composed of numerous protein components containing glutamines and prolines [352,353,354]. Gliadins are mainly monomeric proteins with a molecular weight of approximately 28,000–55,000. They can be classified according to their different primary structures into α/β, γ, and ω types. The glutenin fraction is composed of aggregated proteins linked between chain disulfide bonds. To obtain the glutein network, gliadins and glutenins interact with each other to form covalent and non-covalent bonds. The glutein network contains disulfide bridges (S=S bonds), which are formed by cysteine residues from the same protein complex (intrachain S=S bonds) or different protein complexes (interchain S=S bonds). Gliadins form intrachain disulfide bonds, and glutenins participate in the formation of intra- and interchain S=S bonds [352,355].

Glutein is a cheap, renewable resource that is abundantly available. Glutein fibers are characterized by good mechanical properties and have better tensile properties than biomaterials based on soy protein and casein. In addition, they have water resistance similar to PLA fibers in weakly alkaline conditions and show slightly lower resistance in weakly acidic conditions at high temperatures [356].

Mohamed et al. examined the interaction between poly(lactic acid) and wheat gluten (gluten) using various research techniques, i.e., differential scanning calorimetry (DSC), thermogravimetric analysis (TGA), thermogravimetric spectroscopy, Fourier transform infrared spectroscopy (FTIR), X-ray diffraction, X-ray diffraction, etc. [357]. Hajikhani et al. focused on the fabrication and characterization of a gluten film that was reinforced with electrospun lycopene-loaded poly(lactic acid) nanofibers. Tests carried out on gluten/PLA foil with lycopene encapsulation in PLA nanofibers have a much lower release rate. The research results are beneficial for active food packaging because it allows food to be stored in the packaging for longer [358]. Recently, there has been increasing interest in the development of biopolymer films and coatings using protein, polysaccharide and lipid materials. Ghorpade et al. examined the mechanical and barrier properties of the obtained wheat gluten film coated with poly(lactic acid). PLA-coated foils were more resistant to stretching and were resistant to water vapor penetration [359].

Cho et al. developed and characterized glycerol-plasticized wheat gluten (WG) pressure-molded laminates supported by PLA films. This research confirmed that lamination increased the strength of the foil with the addition of wheat gluten. Very good oxygen barrier properties were demonstrated by laminates with a lower content of a glycerol plasticizer. It was confirmed that the PLA layer also prevented the loss of the glycerol plasticizer from the WG layer. The obtained fully renewable materials which were characterized by a high gas tightness, with sufficient mechanical integrity, with an extrusion coating, and with potential applications in paper/board [360].

### 5.7. Keratin

The biological properties, mechanical durability and excellent biocompatibility of keratin-based biomaterials have attracted intensive research over the last few decades. The complex three-dimensional structure of keratin, unlike other natural polymers, e.g., collagen, starch, chitosan, means that keratin requires the use of difficult chemical conditions for dissolution and extraction. Keratin has found wide application in biomedical fields. It is a component of hair, wool, feathers, and nails [361,362]. Keratin proteins, through self-assembly, create fibers with four levels of structure, and the skeleton is formed by polypeptide chains. Although up to 20 amino acids can form keratins, its main components are alanines and glycines [363].

Keratin is composed of two types: alpha (α)-keratin and beta (β)-keratin, each of which has similar amino acid sequences and biological functions. Filaments are embedded in an amorphous keratin matrix. Keratin-based materials are sensitive to deformation [364]. Tanase et al. developed innovative composites using PLA, chitosan, and keratin. The material was characterized by reduced tensile strength, a significant increase in hardness and good absorption of surface properties with good cell viability/proliferation, which suggested that the composites obtained in the research may have potential applications in medicine [365]. Rojas-Martínez et al. obtained PLA composite scaffolds reinforced with keratin and chitosan, using 3D printing. The analysis of the results provided evidence of the influence of the reinforcement size on the tested properties [366]. The research group of Sanchez-Olivares et al. used keratin fibers from tanning industry waste to prepare completely ecological materials based on PLA composites. The mechanical properties of the material were improved and completely ecological polymer composites were easily prepared [367].

Disposing of waste feathers is expensive and complicated. Ertek et al. proposed an innovative valorization of recycled chicken feather keratin in combination with PLA. Keratin is a component that can be used in various industries, including: medical, food, cosmetic, or agricultural. The thermomechanical, antibacterial, and wetting properties of the surface of electrospun films were influenced by the size of the formed nanofibers, which were adjusted by the keratin content. The research results suggested that the created bio-based materials may be a material alternative that is more environmentally friendly, recycled and can be used in ecological food packaging [368].

### 5.8. Casein

**Figure 27:**
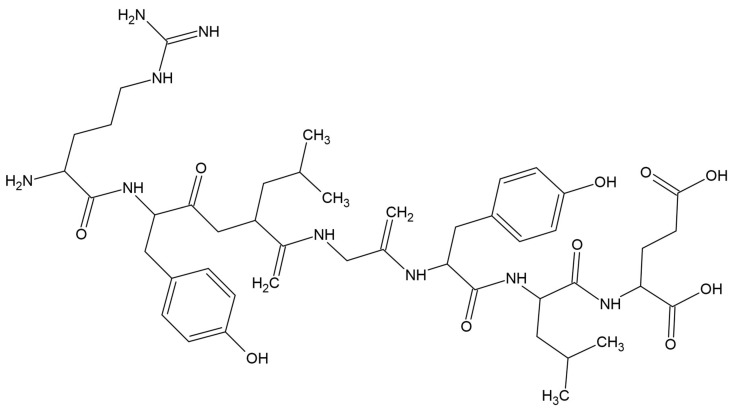
The chemical structure of casein.

Caseins (Figure 27) are proteins, which have an open and flexible conformation [369]. There are four main casein molecules: α-_S1_, α-_S2_, β, and κ-casein, which differ in their amino acid sequences and interactions with hydrophobic and hydrophilic ingredients. It is a cheap, abundant, and easy-to-modify protein found, for example, in milk. Due to its high affinity for hydrophobic substances and its generally safe properties, it can be used in the creation of micro- and nano-capsules [370].

The ability of casein molecules to bind calcium phosphate is achieved by its phosphorylation. Glycosylation of 50% of κ-casein renders its C-terminal portion hydrophilic, being important for micellar structure and stability. The open and flexible conformations of casein confer prolyl residues. The high flexibility of casein particles means that they have excellent surface-active and stabilizing properties [371]. Caseins have the ability to interact with numerous bioactive molecules through hydrophobic, hydrophilic, and electrostatic interactions [372].

Zhang et al. prepared fully bio-based, flame-retardant poly(lactic acid) composites containing casein, which was incorporated into a PLA matrix. This research showed that incorporating casein into the PLA matrix can maintain the biodegradable nature and improve the fire resistance of PLA without sacrificing too many mechanical properties. The analyzes confirmed that casein acted in both the condensation and gas phases [373].

Zhang et al. prepared fully bio-based, flame-retardant poly(lactic acid) composites containing casein, which was incorporated into a PLA matrix. This research showed that incorporating casein into the PLA matrix can maintain the biodegradable nature and improve the fire resistance of PLA without sacrificing too many mechanical properties. The analyzes confirmed that casein acted in both the condensation and gas phases [373]. In recent years, there has been interest in fiber-reinforced polymer materials. Popuri et al. developed casein composites as alternative biodegradable polymers, which were characterized by an increase in the hardness of the materials [374].

Yovcheva et al. developed multilayer polyelectrolytes from a combination of natural casein and chitosan polymers, using the layer-by-layer method on pre-charged PLA pads, which were successfully cross-linked. They allowed to obtain a stable and porous structure with lower water capacity and greater potential for use in the biomedical field [375].

Gu and Catchmark’s elaborated cellulose-reinforced PLA composites using casein protein from whole milk as a dispersant. Casein has the potential to interact with the PLA and cellulose matrix. Improved cellulose dispersion, thanks to the presence of casein, ensured better mechanical properties of the material. The affinity of casein for PLA was important for obtaining the strength and stiffness of composites [376].

### 5.9. Insulin

Insulin is composed of 51 amino acids. It has the form of two peptide chains that are connected by disulfide bonds [377]. The **A** chain consists of 21 amino acid residues, while the **B** chain consists of 30 amino acid residues [378]. Insulin is a protein used in the pharmacotherapy of diabetes [379]. In the body, it is mainly secreted by β cells in the islets of Langerhans of the pancreas. It plays a particularly important function in glucose homeostasis, cell growth, and metabolism [380].

Liu et al. prepared PLA/poly(lactic-*co*-glycolic acid) (PLGA)-based microcapsules with recombinant human insulin (rhI) using the membrane emulsification method. The efficiency of encapsulation was dependent on, for example, the PLA/PLGA ratio, the amount of rhI charge, the pH value in the external aqueous phase, the size of microcapsules, etc. The test results indicated that much higher encapsulation efficiency can be obtained compared to the mechanical mixing method [381].

The stability of insulin in a mixture of poly(L-lactide) and polyethylene glycol (PEG) polymers, which form biodegradable microparticles, was studied by Yeh. Insulin was successfully trapped in microparticles formed by PLA and PEG. The use of PLA/PEG polymer blends resulted in a stable morphology of the microparticles and reduced fragmentation and aggregation of the associated insulin [382]. Slager and Domb developed new insulin-PLA complexes using the stereocomplexation phenomenon. The materials showed sustained insulin release. The obtained macromolecular stereocomplexes, thanks to molecular complexation with enantiomeric polymers, may be a potential beginning of the development of a new generation of systems for the controlled release of peptides and proteins [383]. Kwong et al. discovered insulin delivery via sustained release of the hormone from a biodegradable polymer matrix, poly(l-lactic acid) (PLA), using emulsion/solvent evaporation and solvent casting techniques, respectively. The insulin-PLA granules showed relatively little insulin burst effect in vitro and showed an almost constant insulin release rate during the first hours. In animal studies, insulin-PLA preparations successfully lowered blood glucose levels [384]. Elvassore et al. developed insulin-loaded poly(ethylene glycol)/poly(lactide) (PEG/PLA) nanoparticle materials using anti-solvent techniques. This process offered a way to produce NPs containing proteins that were necessary for practical applications including pharmaceuticals. The results showed that the addition of PEG in the formulation played an important role in the phenomenon of simultaneous precipitation of the solute, as well as in determining the release behavior and chemical-physical properties of the formulation [385]. Ma et al. conducted preliminary tests of the material based on microencapsulated polylactide insulin in vitro and in vivo. This research showed that polylactide microcapsules were able to protect insulin against proteolytic degradation in the gastrointestinal tract. The obtained polylactide microcapsules can act as a carrier for oral administration of insulin [386].

## 6. Summary of the Properties and Application of PLA Systems with Biomolecules

1. The wide scope of combining PLA with biomolecules in order to create biomaterial conjugates was discussed in this review. The method of connection of different structures affects the properties and functions of bioconjugates. Conjugation techniques to create biomaterials have provided the versatility necessary to tune the structure and functions of a given biomolecule-polymer conjugates for desired applications. Blending of PLA with other polymers (or biomolecules) and different additives is easier and simpler modification method than copolymerization of lactide with various monomers and grafting additives on a chain of PLA.
2. The interest in PLA-based conjugates has resulted in the development of their preparation and has enabled the production of increasingly diverse and useful materials with tailored biological, mechanical, and chemical properties to best imitate the properties of the natural extracellular matrix. As a result, important new classes of materials have been developed that are environmentally sensitive, responding to the biological environment, including the release or degradation of cell-required growth factor, which may result in increased effectiveness of materials as tissue substitutes.
3. It is expected that in the future the conjugation of PLA and biomolecules will continue to evolve and adapt materials and their properties through the development of chemical and biological methods of biomaterial synthesis, allowing for better control of the emerging structure.
4. Advances in polymeric materials should facilitate synthesis of new generations of PLA-derived materials with controlled functions, offering expanded possibilities in tissue replacement and drug delivery applications.
5. The biobased and biodegradable PLA with low immunogenicity, non-toxicity, and good mechanical properties, has found numerous pharmaceutical and biomedical applications. It can be easily processed using injection molding and 3D printing, which is very useful for the fabrication of complex structures of implants and orthopedic medical devices. Due to its biocompatibility, biodegradability, mechanical strength, processability, and self-assembly of surface microstructure of PLA materials finds a wide range of biomedical applications: for a drug delivery, wound management, cell culturing, tissue engineering, and tumor therapy [387,388,389].
6. Other copolymers or blends of PLA-based biodegradable materials, such as PLA/polyglycolide acid (PGA), PLA/poly(3-hydroxybutyrate-*co*-valerate) PHBV, PLA/polycaprolactone (PCL), PLA/starch showed improved properties for biomedical applications. Biomedical grade poly(L-lactide) (PLLA) was most often used biodegradable or bioabsorbable biopolymer for biomedical devices [141,390].
7. A large number of biomolecules was used for modifications of PLA properties and results in each case depend on a kind of biomolecule and modification conditions. More research is still needed to fully realize the potential of PLA combined with biomolecules and it will mainly focus on improvement of PLA’s toughness, flexibility, and heat resistance, making it more versatile and suitable for a wider range of applications. Future studies should also lead to further improvement in properties of PLA and growing biomedical applications of PLA modified with polysaccharides, proteins, and nucleic acids. However, at present it is difficult to predict the future practical applications of PLA functionalized with biomolecules [388,391].

## Data Availability

The data are included in the text.
